# Direct Observation of Hybridization Between Co 3*d* and S 2*p* Electronic Orbits: Moderating Sulfur Covalency to Pre‐Activate Sulfur‐Redox in Lithium–Sulfur Batteries

**DOI:** 10.1002/advs.202412038

**Published:** 2024-12-27

**Authors:** Di Wang, Yaozu Jia, Qi Jin, Fengying Tian, Qiong Gao, Xu Xu, Huiqing Lu, Lili Wu, Xinzhi Ma, Xitian Zhang

**Affiliations:** ^1^ Key Laboratory for Photonic and Electronic Bandgap Materials Ministry of Education School of Physics and Electronic Engineering Harbin Normal University Harbin Heilongjiang 150025 China; ^2^ State Key Laboratory of Advanced Technology for Materials Synthesis and Processing School of Materials Science and Engineering Wuhan University of Technology Wuhan Hubei 430070 China

**Keywords:** d‐band center, durability, inverse photoemission spectroscopy/ultraviolet photoelectron spectroscopy (IPES/UPS), lithium–sulfur batteries, shuttle effect

## Abstract

Lithium–sulfur batteries (LSBs) offer high energy density and environmental benefits hampered by the shuttle effect related to sluggish redox reactions of long‐chain lithium polysulfides (LiPSs). However, the fashion modification of the *d*‐band center in separators is still ineffective, wherein the mechanism understanding always relies on theoretical calculations. This study visibly probed the evolution of the Co 3*d*‐band center during charge and discharge using advanced inverse photoemission spectroscopy/ultraviolet photoemission spectroscopy (IPES/UPS), which offers reliable evidence and are consistent well with theoretical calculations. This, coupled with in situ Raman and X‐ray diffraction (XRD) and electrochemical data, co‐evidences a novel pre‐activating S redox mechanism in LSBs: LiPSs desert/insert in C‐N matrixes within a series of Co@NCNT‐based separators. The insight of the S redox pre‐activation is discovered that the Co 3*d*‐band center downshifts to hybridized with S 2*p* orbitals in LiPSs, giving rise to a more pronounced S covalency and thus accelerating the conversion of LiPSs to S₈. Benefiting from these advantages, the optimized LSB possesses a minimal decay rate of 0.0058% after 200 cycles at a high discharge rate of 10 C. This study provides new insights into LSB mechanisms and supports conventional theoretical models of the *d*‐band center's impact on LSB performance.

## Graphical abstract

Advanced inverse photoemission spectroscopy/ultraviolet photoelectron spectroscopy (IPES/UPS) experimentally probes the hybridization of Co 3*d* and S 2*p* after Li (from LiPSs) insertion in NC matrix, which gives rise to an enhanced sulfur covalency can pre‐activate the sulfur‐redox and thus improves lithium–sulfur battery performances.

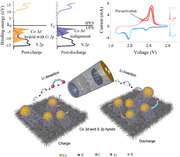

## 1 Introduction

The increasing demand for advanced batteries with long cycle life, high capacity, and low self‐discharge rates is driven by the development of vehicles and portable electronic devices.^[^ ^1^, ^2^ ^]^ Lithium–sulfur batteries (LSBs), with a theoretical energy density of 2600 Wh kg^−1^ and a specific capacity of 1675 mAh g^−1^, are considered next‐generation energy storage devices due to their environmental friendliness, abundant resources, and high safety.^[^ ^3^, ^4^ ^]^ However, their commercial potential is still limited by several challenges. Notably, 1) the relatively high overpotential during discharge and charge processes, which arises from electrode chemical reactions,^[^ ^5^ ^]^ and 2) the shuttle effect, where intermediate lithium polysulfides (LiPSs), specifically long‐chain Li_2_S_n_ (6 ≤ n ≤ 8), penetrate the separator, leading to deposition on the cathode, reduced capacity, and shortened cycle life.^[^ ^6^, ^7^ ^]^

These challenges are typically mitigated by employing well‐conducting metal centers to absorb and convert sulfur species, achieved through the structural regulation of LSBs components (i.e., cathode, anode, electrolyte, and separator).^[^ ^8^, ^9^, ^10^, ^11^ ^]^ Functionalizing the separator has emerged as one of the most effective strategies, with porous materials offering significant benefits for sulfur redox and ion transport.^[^ ^12^, ^13^, ^14^ ^]^ For example, cobalt nitrogen‐doped mesoporous carbon (Co‐N‐MC) not only facilitates efficient electron transfer but also provides a confined space for LiPSs redox reactions.^[^ ^15^ ^]^ However, the weak polarity of Co‐N‐MC primarily restricts sulfur through a physical barrier, making it challenging to fully suppress the shuttle effect. In contrast, metal‐organic framework (MOF)‐derived metal‐nitrogen‐carbon materials exhibit excellent polarity modulation in addition to their intrinsic mesoporous characteristics.^[^ ^16^, ^17^ ^]^ Despite this, their influence is generally limited to the initial chemical absorption of LiPSs, and they struggle to enhance subsequent LiPSs conversions. This limitation constrains shuttle effect inhibition and limits the discharging/charging rate. Therefore, there is a critical need to develop novel and effective strategies to activate sulfur redox processes.

Li⁺ insertion and desertion in materials with unique layered structures or interplanar spacings larger than the Li‐ion radius endow lithium‐ion batteries (LIBs) with favorable energy and power densities.^[^ ^18^, ^19^ ^]^ It is, therefore, reasonable to expect that Li⁺ insertion/stripping in LiPS or the electrolyte could improve the redox capacity of sulfur in LiPSs by moderating the sulfur redox process. This enhancement would result in increased power densities, energy density, and extended cycle life. Additionally, Li⁺ insertion/desertion could provide LSBs with extra capacities through the conventional charge/discharge mechanism. Moreover, studies have shown that the Li⁺ insertion/desertion capability is influenced by the structural reorganization of the host materials,^[^ ^20^ ^]^ a phenomenon that is size‐dependent.^[^ ^21^, ^22^ ^]^ Therefore, integrating the Li⁺ insertion/desertion mechanism into LSBs and synergizing it with conventional metal‐dominated sulfur redox pathways presents a promising strategy for enhancing LSBs performance. However, this approach remains underexplored.

In this study, a secondary carbon cloth (CC) separator was fabricated by coating ZIF‐67‐derived cobalt‐inserted nitrogen‐containing carbon nanotube arrays (Co@NCNT‐CC) with particle sizes of ≈5, 10, and 20 nm. Advanced characterization techniques, including ultraviolet photoelectron spectroscopy (UPS), inverse photoelectron spectroscopy (IPES), in situ Raman spectroscopy, X‐ray diffraction (XRD), and electrochemical cyclic voltammetry (CV), were employed to systematically investigate the mechanisms of Li⁺ insertion/dissolution, their dependence on particle size, and the resulting benefits in LSBs. The study revealed that the Li⁺ insertion/dissolution mechanisms significantly enhance the sulfur redox process, with a pronounced dependence on particle size. Specifically, smaller particle sizes were found to increase the bandgap to an optimized value, thereby improving the absorption of sulfur species and facilitating subsequent redox reactions. Leveraging this advanced mechanism, the LSBs exhibited energy densities of 256.4 mAh g^−1^ at high discharge/charge rates of 10 C, respectively.

## 2 Results and Discussion

**Figure 1:**
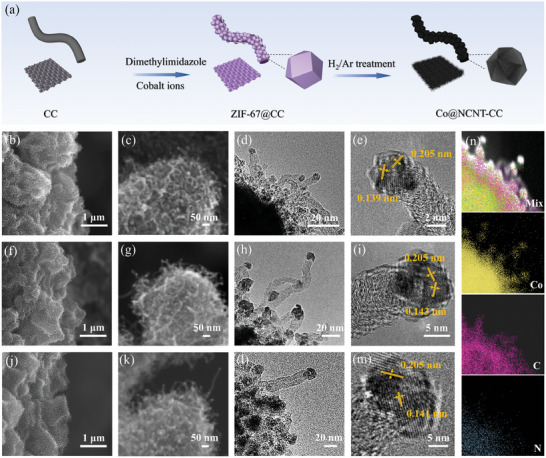
Schematic diagram and structural characterization of the Co@NCNT‐based materials. a) Diagram of the synthetic process. Morphologic and structural information for Co@NCNT‐5, Co@NCNT‐10, and Co@NCNT‐20, b,f,j) SEM image, c,g,k) High‐magnification SEM images; d,h,l) TEM images, e,i,m) High‐magnification TEM images. n) Elemental mappings of Co@NCNT‐5.

The synthesis procedure of these catalysts is illustrated in **Figure** **1**a. Initially, orthododecahedral ZIF‐67 was synthesized on CC using a classic thermostatic hydrothermal method (procedure I).^[^ ^23^ ^]^ Subsequently, the obtained material underwent H_2_/Ar treatments at 435 °C with different flow rates (procedure II, 300, 320, and 340 sccm, respectively). Finally, the Co@CNT‐CC catalyst was obtained after cooling down to room temperature.^[^ ^24^ ^]^ Scanning electron microscopy (SEM) was utilized to characterize the morphologies of these products. It was observed that after procedure I, the CC was entirely covered by a smoothed substance on the surface (Figure S2a–c, Supporting Information), which retained its original morphology but became rough after procedure II (Figure S3, Supporting Information). Corresponding XRD data suggests that the products of procedure I exhibit an orthododecahedron‐like structure of ZIF‐67, which fully converts to cobalt along with an amorphous carbon substance after H_2_/Ar treatment (Figure S4, Supporting Information). High‐magnification SEM images illustrate that the orthododecahedron structure slightly withers and becomes covered with dense nanowires externally (Figure 1b,c,f,g,j,k). The lengths of the nanowires obtained from different H_2_/Ar flow rates were calculated to be ≈50 nm based on further enlarged SEM images. Corresponding TEM images revealed that these nanowires are hollow, with each nanofiber containing a nanoparticle inserted in its tip (Figure 1d,h,l). The diameters of both the nanoparticle and nanofiber diameters increase with the H_2_/Ar flow rates. For instance, as depicted in high‐resolution TEM (HRTEM) images (Figure 1e,i,m), their diameters were calculated to be ≈5, 10, and 20 nm when the flow rates were 300, 320, and 340 ppm, respectively. The lattice parameters for these samples were calculated to be 0.205 and 0.139 nm, 0.205 and 0.143 nm, and 0.205 and 0.141 nm, which correspond well with the (111) and (220) planes of metallic Co, respectively. This was further confirmed by their corresponding Selected area electron diffraction data (Figure S5, Supporting Information), which showed significant diffraction point matrices. Elemental mappings for these samples were characterized subsequently. From Figure 1n, it is evident that the cobalt particles at the tips of the carbon nanotubes contain two elements, Co and N, and are distributed more uniformly, Co@NCNT‐10 (Figure S6a, Supporting Information), Co@NCNT‐20 (Figure S6b, Supporting Information). Additionally, element C has a more uniform distribution on the carbon nanotubes. These data suggest that the Co@NCNT‐CC has been successfully synthesized.

**Figure 2:**
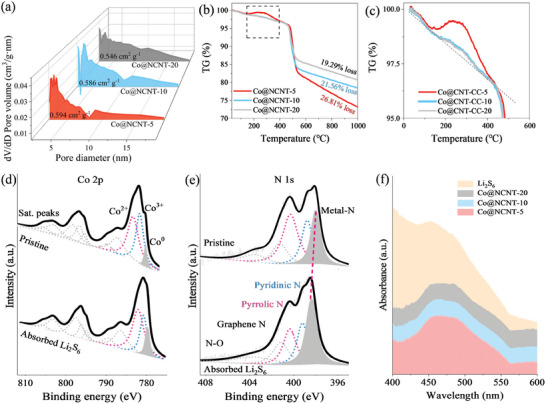
Physical properties of Co@NCNT‐based materials. a) Pore size distribution diagram; b,c) Thermogravimetric analysis diagram; High‐resolution XPS spectrum of Co@NCNT‐5, d) Co 2*p*, e) N 1*s*; f) UV–vis spectra after Li_2_S_6_ absorption.

Co@NCNT‐5 has a larger pore volume of 0.594 cm^2^ g^−1^ compared to Co@NCNT‐10 (0.586 cm^2^ g^−1^), Co@NCNT‐20 (0.546 cm^2^ g^−1^) (**Figure** **2**a) and has a superior comparative area of 226.80 m^2^ g^−1^, Co@NCNT‐10 (206.00 m^2^ g^−1^), Co@NCNT‐20 (201.52 m^2^ g^−1^). Figure 2b shows the thermogravimetric curves, and it is obvious that the thermogravimetric curves of Co@NCNT‐5, 10, and 20 show a significant bulge in the range of 180–400 °C. It is pronounced for Co@NCNT‐5, which suggests that pre‐oxidation occurs during the reaction process and makes S–S coupling (Figure 2b,c). To serve as a separator in LSBs, it is crucial for materials to possess strong capabilities for S absorption and mechanical durability. The adsorption abilities toward polysulfides of the Co@NCNT‐CC‐*x* samples were evaluated in 0.01 m Li_2_S_6_ solutions. Figure S6 (Supporting Information) illustrates that the Li_2_S_6_ solution cleared within 3 h after introducing Co@NCNT‐5, while for Co@NCNT‐10 and Co@NCNT‐20, it took more than 3.5 and 4 h, respectively (Figures S7 and S8, Supporting Information). Besides the observable macroscopic color change, the adsorbed products were also analyzed using a UV–vis spectrophotometer. The corresponding UV–vis spectrum indicates that the intensities of the Li_2_S_6_ absorption peaks follow a trend of Co@NCNT‐5 < Co@NCNT‐10 < Co@NCNT‐20 (Figure 2f), suggesting that a smaller diameter of Co@NCNT‐CC‐*x* correlates with enhanced Li_2_S_6_ absorption capability. Additionally, the positions of the Li_2_S_6_ peak for these samples exhibit a redshift as the diameter decreases, indicating that a smaller Co nanoparticle diameter leads to an abnormal absorbed pathway between Co@NCNT‐CC‐*x* and Li_2_S_6_.

The chemical composition of the products before and after the absorption of Li_2_S_6_ was characterized using X‐ray photoelectron spectroscopy (XPS). Figure S9 (Supporting Information) displays the survey illustrating that the pristine Co@CNT‐CC‐*x* sample contains Co, N, and C elements. In the high‐resolution Co 2*p* and N 1*s* spectral regions of the Co@NCNT‐5 material in Figure 2d,e, the Metal‐N gradually increases and shifts while the peak of Co^3+^ steadily decreases upon contact with Li_2_S_6_. Combining this shift of the Metal‐N binding energy, it can be inferred that Li^+^ may originate from LiPSs, and Li^+^ is inserted in the vicinity of the S‐oxide. This is attributed to the presence of numerous defects in the material, causing Li^+^ to occupy defective positions of the pyrrole nitrogen. Additionally, Co ions can adsorb Li^+^, exerting a pulling effect on the Metal‐N bonding and achieving the effect of Li^+^ inserting. The XPS plots of C 1*s* before and after adsorption are depicted in Figure S10 (Supporting Information). In Figure S11 (Supporting Information), the binding energies and peak intensities of Co and C in Co@NCNT‐10 remain essentially unchanged before and after the adsorption of Metal‐N. However, the intensities of graphitized N are significantly decreased. In Figure S12 (Supporting Information), the XPS spectra of Co@NCNT‐20 reveal substantial shifts in both Co 2*p* and N 1*s*. Further analysis of these materials leads to the conclusion that when used as an intercalation layer in LSBs, Co@NCNT‐CC exhibits a greater degree of defects and more atomic crystal defects. This, in turn, results in higher electrode cycling efficiency of the batteries and achieves a new strategy of S redox pre‐activation.^[^ ^25^, ^26^ ^]^

**Figure 3:**
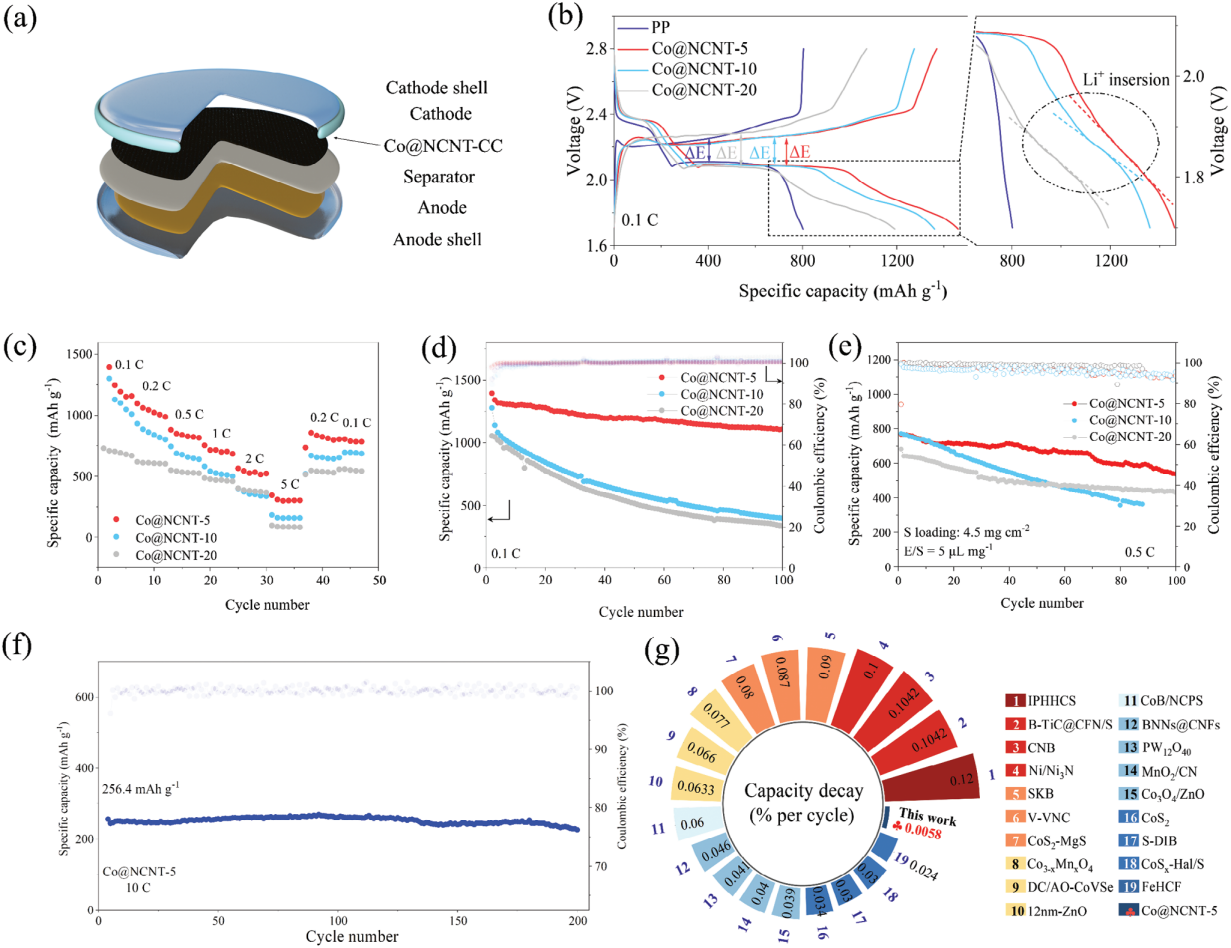
LSB performances. a) Battery assembly diagram; b) GCD curves; c) magnification diagram; d) Cycle diagram at 0.1 C magnification; e) Cycle diagram of LSBs under high‐loading and lean electrolyte at 0.5 C magnification; f) Long cycle diagram at 10 C magnification; g) Performance comparison diagram.

Based on their excellent mechanical strength and toughness that can recover after multiple folding shown in Figure S13 (Supporting Information), the LSBs were assembled, as shown in **Figure** **3**a. Figure 3b displays the constant‐current galvanostatic charge–discharge (GCD) curves of the LSBs at a charge–discharge rate of 0.1 C (1 C = 1675 mAh g^−1^). Notably, a third discharge plateau is observed in the GCD curve. This arises from the Li⁺ insertion during discharge, which is reflected as an oxidation peak in the corresponding CV curve, contributing additional capacity.^[^ ^27^ ^]^ In our case, the fresh CC contributes 88 mAh g^−1^ (Figure S14a, Supporting Information), which is irreversibly manifested by the capacity difference between charge and discharge (Figure S14b,c, Supporting Information). Co@NCNT‐5 has superior multiplicity performance and still allows capacity recovery to 990 mAh g^−1^ during recovery from 5 C to 0.2 C (Figure 3c). The capacities of LSBs assembled with Co@NCNT‐5, Co@NCNT‐10, and Co@NCNT‐20 are 1394, 1277, and 1054 mAh g^−1^, respectively (Figure 3d). Clearly, LSBs assembled with Co@NCNT‐5 outperform others across discharge rates ranging from 0.2 to 2 C (Figure S15, Supporting Information). It is noteworthy that the discharge capacity increases with decreasing diameter of Co@NCNT. After undergoing cycling from 0.1 to 5 C, the Co@NCNT‐5 coupled LSBs exhibit an impressive 64.7% capacity recovery (856.6 mAh g^−1^), exceeding that of LSBs coupled with Co@NCNT‐10 (669.5 mAh g^−1^) and Co@NCNT‐20 (546.5 mAh g^−1^). Moreover, the Co@NCNT‐5 coupled LSBs maintain capacities of 1194.5 and 1108.0 mAh g^−1^ after 50 and 100 discharge/charge cycles, respectively, significantly surpassing LSBs coupled with Co@NCNT‐10 (591.9 and 395.7 mAh g^−1^), and Co@NCNT‐20 (515.9 and 335.7 mAh g^−1^). The LSB performance was evaluated under challenging conditions, including high sulfur loading and lean electrolytes. As shown in Figure 3e, the LSB paired with Co@NCNT‐5 delivers an initial capacity of 783 mAh g^−1^ and an exceptionally low capacity decay rate of 0.312% over 100 cycles. This performance is markedly superior to that of LSBs paired with Co@NCNT‐10 (772 mAh g^−1^, 0.528%) and Co@NCNT‐20 (682 mAh g^−1^, 0.369%), respectively.

It is noteworthy that Co@NCNT‐5 enables the LSB to achieve an exceptionally high discharge/charge rate of 10 C, coupled with an ultra‐low capacity decay rate of just 0.0058%, maintaining a capacity of 226 mAh g^−1^ after 200 cycles (Figure 3f). This performance is comparable to, or even surpasses, that of many advanced benchmark materials (Figure 3g).^[^ ^28^, ^29^, ^30^, ^31^, ^32^, ^33^, ^34^, ^35^, ^36^, ^37^, ^38^, ^39^, ^40^, ^41^, ^42^, ^43^, ^44^, ^45^, ^46^ ^]^ Capitalizing on these strengths, the Co@NCNT‐5 coupled LSBs efficiently powered red and yellow light‐emitting diodes, exhibiting minimal luminance degradation even after 5 h, surpassing LSBs coupled with Co@NCNT‐10, Co@NCNT‐20, as well as PP (Figure S16, Supporting Information). These light‐emitting diode experiments further validate the advantages of integrating Co@NCNT‐CC‐based separators, particularly Co@NCNT‐5. These findings deviate from conventional reports on LIBs, suggesting that Co@NCNT‐CC coupled LSBs possess distinctive discharge/charge mechanisms alongside the well‐known S redox reactions. An in‐depth exploration into the underlying reasons for the enhanced capabilities of Co@NCNT‐CC coupled LSBs and their impact on electrochemical impedance spectroscopy (*EIS*) and cycling performances would be of considerable interest. *EIS* analysis reveals impedance values of 6.6, 8.9, and 9.5 Ω for LSBs coupled with Co@NCNT‐5, Co@NCNT‐10, and Co@NCNT‐20, respectively, all notably lower than the value of 47.65 Ω observed in PP‐coupled LSBs (Figures S17 and S18, Supporting Information). Significantly, a conspicuous relationship between the diameter of Co@NCNT and the charge transfer resistance (*R*_ct_) value becomes evident, with smaller Co@NCNT diameters correlating with decreased *R*_ct_ values. This correlation is further substantiated through subsequent evaluation of the rate performances and durabilities of these LSBs. Consistent with the *EIS* results, Co@NCNT‐5, characterized by the smallest diameter, demonstrates superior rate performance and higher capacities.

**Figure 4:**
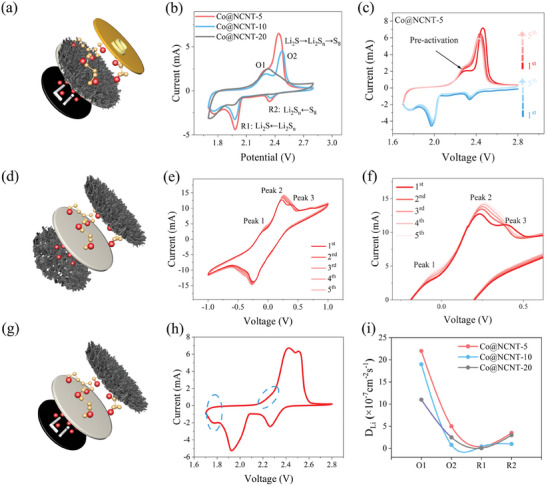
Electrochemical characterizations of the LSBs. a) Schematic of the assembly of a common Co@NCNT‐5 coupled LSB; b) CV images of Co@NCNT‐5, 10, 20; c) CV image of five circles at 0.1 mV s^−1^; d) Assembly diagram of the prepared symmetric CV cell (electrolyte is Li_2_S_6_); e) Symmetric CV diagram; f) Symmetric CV magnification; g) Schematic of LSB assembly with electrolyte Li_2_S_6_; h) CV diagram; i) Li^+^ diffusion diagram.

**Figure** **4**a shows the schematic diagram of the cell assembly of Co@NCNT‐5 coupled LSBs, under which the CV test of the cell was performed in this cell preparation process. At the same scan rate, as the number of turns increases, a small bump peak gradually appears near 2.28 V, and another oxidation peak appears near 2.42 V. The CV of the coupled LSBs is shown in Figure 4b,c, which shows the CV of the coupled LSBs. All LSBs exhibit two pairs of redox peaks, occurring ≈2.3 and 1.98 V (denoted as O_1_ and R_1_, respectively), and 2.4 to 2.5 and 2.35 V (denoted as O_2_ and R_2_, respectively). Remarkably, the O_1_ peaks for these Co@NCNT‐assembled LSBs demonstrate an ≈120 mV blueshift compared to LSBs in other reports, which is unusual to previously reported LSBs.^[^ ^47^, ^48^, ^49^ ^]^ This distinctive phenomenon suggests that the conversion of Li_2_S‐Li_2_S_n_ may undergo pre‐activation upon assembly of the Co@NCNT separator. Here, we speculate that the peak near 2.37 V corresponds to the oxidation process (Li^+^ inserting/deserting), and at 2.42 V, the second oxidation peak is the fusion of the two oxidation peaks.

To further support this hypothesis, we prepared Co@NCNT‐based symmetric and lithium batteries, both using Li_2_S_6_ as the electrolyte. Figure 4d shows a schematic of the symmetric battery. The corresponding CV curves exhibit excellent symmetry and contain three oxidation peaks similar to those mentioned earlier (Figure 4e). In the enlarged CV curve, as cycling progresses, peak 1 intensifies while peaks 2 and 3 merge, consistent with the CV results obtained in our experiments (Figure 4f). Notably, peak 1 appears below 0 V, indicating that its oxidation process is spontaneous and does not require an external electric field. Figure 4g presents a schematic of the Li battery with Co@NCNT as the cathode. In its CV curve, both the oxidation peak 1 and the corresponding reduction peak are present, aligning with the results of the separator modification, and matching the positions of other Li^+^ desertion and insertion peaks. These results demonstrate the occurrence of LiPSs deintercalation in Co@NCNT during the charge and discharge cycles. This hypothesis is further corroborated by the ion mobility data: during the initial oxidation step (Li^+^ desertion/insertion), the mobility is significantly higher than in the subsequent oxidation processes 2 and 3.

As shown in Figure S19 (Supporting Information), Q_H_ and Q_L_ respectively represent the capacities of the high‐voltage and low‐voltage discharge platforms, which can be calculated from the GCD curves. The ratio of Q_H_ to Q_L_ serves as a quantitative indicator to assess the electrocatalytic capability of the host material in converting LiPSs. Unlike previous cases where only two discharge platforms were observed, the batteries in this experiment also exhibit a third discharge platform (contributing to secondary capacity). This signifies the unusual discharge mechanism observed in this experiment. Furthermore, notably, Co@NCNT‐5 demonstrates higher Q_H_, Q_L_, and a higher Q_L_/Q_H_ ratio, indicating its outstanding electrocatalytic performance. Figure S20 (Supporting Information) illustrates the differential CV curves of Co@NCNT‐5. The baseline voltages and current densities are defined as the values before the appearance of redox peaks, where the point with the smallest change in current density is denoted as dI/dV = 0. The corresponding onset potentials of the two reduction peaks I, II, and the corresponding oxidation peak III are accordingly indicated. The baseline voltage of Co@NCNT‐5 in Figure S20b (Supporting Information) corresponds to Figure S20a (Supporting Information). Compared to Co@NCNT‐10, 20, Co@NCNT‐5 exhibits a smaller charging overpotential, suggesting pre‐activation, which facilitates the early conversion of Li_2_S to S_8_.

Moreover, for these Co@NCNT‐assembled LSBs, their O_2_ peak shifts negatively, while the R_2_ and R_1_ peaks shift positively, with a smaller diameter a more pronounced peak shift. Furthermore, the corresponding capacity for these redox peaks decreases as the diameter of Co@NCNT decreases. The dependence of peak voltage and capacity on the diameter of Co@NCNT can be evidenced by their corresponding Tafel slopes, where the values follow a pattern of Co@NCNT‐5 < Co@NCNT‐10 < Co@NCNT‐20 (Figure S21, Supporting Information). Similar trends were observed in the activation energies during both the charge (from Li_2_S to Li_2_S_n_) and discharge processes (from S_8_ to Li_2_S). Particularly, the energy gap (*Δ*E) of progress R_1_ (from Li_2_S_n_ to Li_2_S) is much smaller than R_2_ (from S_8_ to Li_2_S_n_), suggesting that our Co@NCNT mainly affects the final discharge product. Furthermore, we observe that a smaller Co diameter corresponds to a smaller energy gap, particularly indicating an increased advantage with a smaller Co@NCNT diameter (Figure S22, Supporting Information). Hence, we suggest that a smaller diameter of Co@NCNT better facilitates the S redox process due to its inherently more favorable thermodynamic energy. By analyzing the CV curves of Co@NCNT‐5, 10, and 20 at different sweep speeds, we can deduce the characteristic of Li^+^ diffusion ($D_{Li^{+}}$), as shown in Figure 4i. Throughout the O_1_, O_2_, R_1_, and R_2_ reactions, the Co@NCNT‐5 cell exhibits a higher $D_{Li^{+}}$ value, indicating improved diffusion kinetics of lithium ions. This facilitates the rapid redox reaction kinetics of S species.

To delve deeper into the reaction kinetics, symmetric CV tests were conducted for Co@NCNT‐5, 10, and 20, as illustrated in Figure S23 (Supporting Information) respectively. The results unmistakably reveal that Co@NCNT‐5 exhibits remarkable cyclic reversibility and symmetry, with slightly larger slopes in its symmetric CV curves compared to the other two. Conversely, Co@NCNT‐10 and 20 demonstrate asymmetry at the outset of the symmetric CV test, indicating inferior cyclic reversibility.

(1) $$I_{p}=\left(2.69\times 10^{5}\right)n^{3/2}v^{1/2}AD_{Li^{+}}^{1/2}C_{Li^{+}}$$

where *I_p_* is the peak current density, *n* is the number of electrons transferred during the redox process, *v* is the scan rate, *A* is the electrode area, $D_{Li^{+}}$ is the Li^+^ diffusion coefficient, and $C_{Li^{+}}$ is the molar concentration of Li^+^. As depicted in Figure S24 (Supporting Information), Co@NCNT‐5 demonstrates superior Li_2_S deposition performance compared to the other two samples. Subsequent evaluations of the Li_2_S deposition characteristics revealed that Co@NCNT‐5 exhibits the highest peak current of 0.19 mA, the shortest nucleation time, and the largest deposition capacity. These findings suggest that, in comparison to Co@NCNT‐10 and Co@NCNT‐20, Li_2_S deposition on Co@NCNT‐5 is significantly accelerated. This indicates that Co@NCNT‐5 effectively reduces the Li_2_S nucleation overpotential and maximally accelerates the nucleation reaction kinetics.

**Figure 5:**
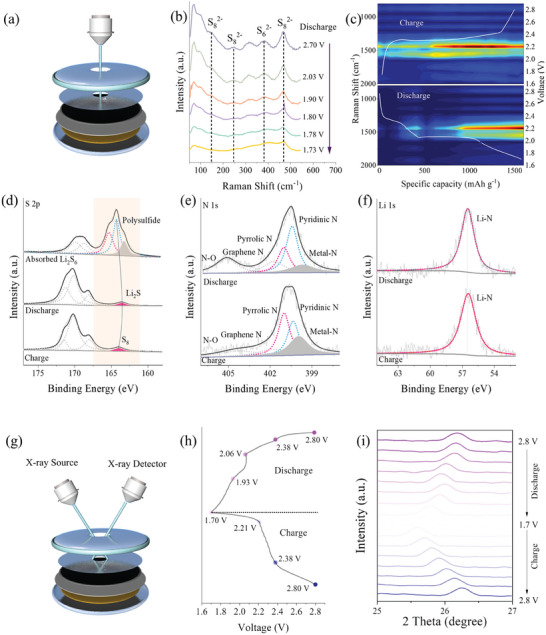
Mechanism discussions based on in situ and advanced techniques. a) Schematic diagram of in situ Raman cell assembly; b,c) In situ Raman analysis with different ranges; XPS spectra of Co@NCNT‐5‐LSB post‐discharging/charging d) S 2*p*; e) N 1*s*; f) Li 1*s*; g) Schematic diagram of an in situ XRD cell; h) GCD curves; i) In situ XRD plot.

**Figure** **5**a depicts a schematic of the in situ Raman analysis setup for the positive electrode. The in situ Raman map of the cell discharge process, with Raman shift in the range of 0–550 cm^−1^ alongside voltage variation, reveals a gradual reduction in the prominence of the S_8_^2−^ peak as the discharge process voltage decreases (Figure 5b). Figure 5c shows contour plots of in situ Raman data, with charging and discharging platforms as references. It is evident that the material experiences increased defect intensity during charging and discharging, indicating the generation of defects during the reaction process. Figure S25 (Supporting Information) displays the Raman spectra of Co@NCNT‐5, 10, and 20, where the D and G peaks are clearly visible with shifted peak positions, indicating structural changes in the materials. Calculations reveal that the I_D_/I_G_ values for Co@NCNT‐5, 10, and 20 are 0.95, 0.90, and 0.88, respectively. The highest I_D_/I_G_ value of Co@NCNT‐5 suggests the highest defect content in its structure during charging and discharging.^[^ ^50^, ^51^ ^]^ Combining these electrochemical results, we can infer that the carbon matrix in the material participates in the reaction during charging and discharging and may be a primary reason for the observed “pre‐activation”.

The S 2*p* spectrum of Co@NCNT‐5 exhibited multiple polysulfide peaks with significant intensities between 163.04 and 165.59 eV following interaction with Li_2_S_6_. During discharge, these peaks were substantially weakened, and after charging, only S_8_ peaks remained, indicating a notable reduction in peak intensity. This observation demonstrates the effective suppression of polysulfides by Co@NCNT‐5. Additionally, peaks corresponding to electrolyte substances within the 167 to 175 eV range remained stable throughout the charge and discharge processes (Figure 5d). Figure 5e reveals that during charging, Li^+^ desertion occurs, leading to the gradual exposure of metal‐N bonds. Conversely, during discharge, Li^+^ penetrates the material, reducing the number of metal‐N bonds, with Li^+^ desertion from pyridine nitrogen and insertion into pyrrole nitrogen sites. In Figure 5f, the Li‐N peak position in the Li 1*s* spectrum remained essentially unchanged before and after charging and discharging, corroborating the previous findings that Co adsorbs Li^+^, forming Li─N bonds with nitrogen.^[^ ^52^ ^]^ As Li^+^ begins to insert into the lattice material, some ions remain trapped, supporting the overall structure. This Li^+^ desertion/insertion mechanism significantly enhances the performance of LSBs assembled with Co@NCNT‐5. Following 100 cycles, Co@NCNT‐5, Co@NCNT‐10, and Co@NCNT‐20 coupled LSBs were dismantled, and the lithium flakes were individually examined. The lithium flakes within the Co@NCNT‐5 coupled LSBs appeared bright and polished, whereas those from Co@NCNT‐10 and Co@NCNT‐20 coupled LSBs exhibited varying degrees of discoloration. This observation suggests that Co@NCNT‐5 effectively mitigates the shuttling effect (Figure S26, Supporting Information). Figure 5g presents a schematic of the in situ XRD unit, with analysis conducted in the 2θ = 25° – 27° range, revealing characteristic carbon peaks (Figure 5i). During discharge from 2.8 to 1.7 V, the 2θ value shifts to a lower angle, indicating lattice expansion due to Li^+^ insertion. Conversely, during charging, the 2θ value shifts higher, reflecting lattice shrinkage as Li^+^ desertion. This confirms the effective Li^+^ insertion and desertion mechanisms.

**Figure 6:**
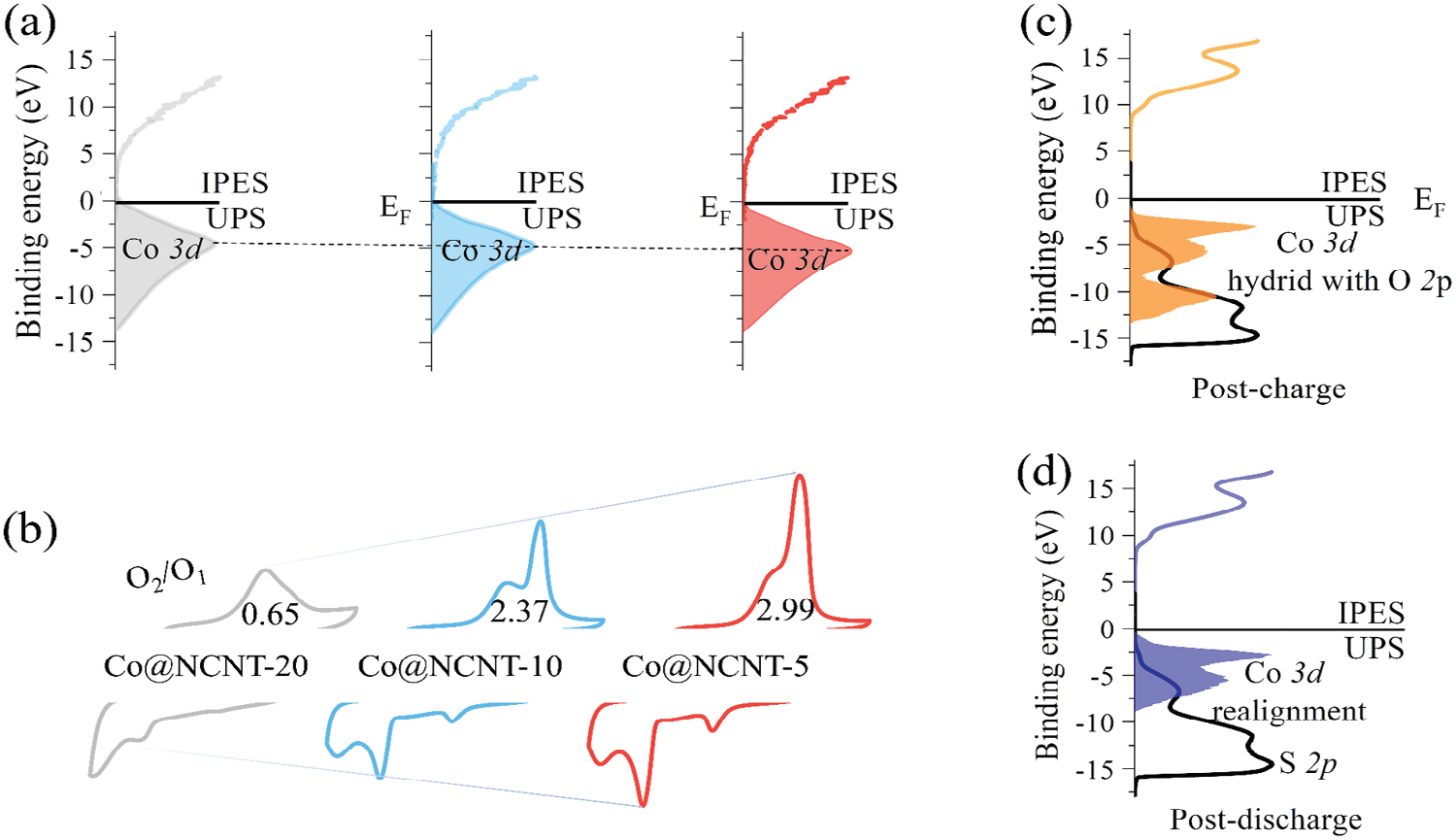
Mechanistic discussion based on IPES/UPS techniques. a) IPES/UPS diagrams for Co@NCNT‐20, 10, 5; b) CV diagram; c,d) Co@NCNT‐5 with its post‐discharge and post‐charge IPES/UPS plots.

Advanced IPES and UPS techniques were utilized to investigate the intrinsic *d*‐band electron distributions, revealing the size‐dependent variations and changes that occur during charging and discharging, thereby elucidating the LSBs mechanism in our study. In the Co@NCNT series, smaller particle sizes cause a more pronounced downward shift in the intrinsic *d*‐band orbitals, with Co@NCNT exhibiting the lowest Co 3*d* orbital *d*‐band center (**Figure** **6**a). This enhances the material's ability to accommodate electrons and hybridize with S 2*p* orbitals.^[^ ^53^, ^54^ ^]^ The correlation between hybridization strength and the charging process is further corroborated by CV analysis, where stronger hybridization is associated with a smoother charging process, as indicated by a larger O₂/O₁ ratio. As illustrated in Figure 6b, the O₂/O₁ values for Co@NCNT‐5, Co@NCNT‐10, and Co@NCNT‐20 decrease from 2.99 to 0.65, corresponding to a progressive decline in *d*‐*p* orbital hybridization. These findings suggest that the presence of hybridization facilitates a smoother charging process, enhances the activation of Li₂S, promotes the cleavage of Li₂S chains to form LiPSs, and facilitates the release of sulfur from lithium binding.^[^ ^55^, ^56^ ^]^

It has been shown that S 2*p* orbitals appeared after the charging processes, which corresponds to the pre‐activation of S in the previous section and the adsorption of Co on S. Meanwhile, the Co *3d* electron orbitals in Co@NCNT are heavily hybridized with the S 2*p* electron orbitals (Figure 6c,d). It is well known that the hybridization of M 3*d* and S 2*p* orbitals leads to covalency between metal and oxygen atoms, giving rise to more intensive to unbound and direct coupling with its adjacent ones.^[^ ^57^, ^58^, ^59^ ^]^ In our LSBs system, once Li‐S is de‐insert in N‐C, S acquires higher covalency as a redox center and coupling between S and S is more likely to occur. Since the S–S coupling barrier is lower than others,^[^ ^60^, ^61^ ^]^ this coincides with the forward shift of the oxidation peak position in the electrochemical CV (Figure 6b). Combine with the pheromone that the Co 3*d* orbital disappeared in the discharging process. It can be confirmed that the separator and S cathode together undergo electron gain and loss during charging/discharging cycles, respectively. This suggests that electrons generated during LiPSs desertion/insertion in NC species are transferred to the Co 3*d* orbitals. This transfer moderates Co‐S covalency, thereby facilitating the charging process from LiPSs to S₈.

**Figure 7:**
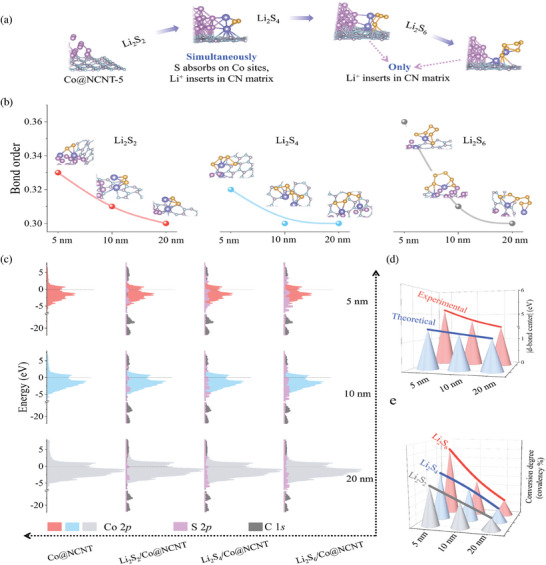
Mechanism discussion based on DFT. a) Co@NCNT‐5 adsorption configurations with Li_2_S_2_, Li_2_S_4_, Li_2_S_6_, respectively; b) S─S bond orders of Li_2_S_2_, Li_2_S_4_, Li_2_S_6_ interacting with Co@NCNT‐5, 10, 20, respectively; c) Partial density of states (PDOS) during charge process for Co@NCNT‐based separator; d) D‐bond center; e) Conversion degree.

To ensure the reliability and accuracy of the data from IPES/UPS and electrochemical CV, theoretical calculations were performed using density functional theory (DFT). We initially simulated the interaction of the discharge product Li₂S with the separator during the charging process. As depicted in **Figure** **7**a, Li₂S₂ adsorbs at the Co center, accompanied by the embedding of Li into the N‐C matrix. This embedding process also occurs during the subsequent transformations into Li₂S₄ and Li₂S₆. A similar process was observed in Co@NCNT‐10 and Co@NCNT‐20 (Figures S27 and S28, Supporting Information). Subsequently, we calculated the bond order (Figure 7b), which quantifies the hybridization strength between the S and S electronic orbitals, that is, the covalent bonding ability, during the conversion of Li₂S₂, Li₂S₄, and Li₂S₆. The results show that, during each LiPSs conversion process, the S─S bond order increases as the particle size of Co@NCNT decreases. Notably, this trend is more pronounced in the sulfur‐rich Li₂S₆ intermediate. Therefore, we conclude that reducing the particle size of Co@NCNT enhances the covalency between S atoms, particularly during the sulfur‐rich phase at the end of the charging process. This suggests that smaller particle sizes facilitate the formation of S─S bonds during the charging process, that is, the charging reaction.

Next, we analyzed the partial density of states (PDOS) of C 1*s*, S 2*p*, and Co 2*p* during the conversion of Li₂S₂, Li₂S₄, and Li₂S₆ in the Co@NCNT series samples. As shown in Figure 7c, there is an overlap between the Co 2*p* and S 2*p* electronic orbitals, but no overlap with the C 1*s* orbitals. This indicates hybridization between Co 2*p* and S 2*p*, but not between Co 2*p* and C 1*s*. Furthermore, we observed that as the particle size of Co@NCNT decreases, the *d*‐band center of the Co 3*d* orbit shifts from −1.141 to −1.254 eV, enhancing the overlap with the S 2*p* electronic orbitals. The strength of this overlap increases as the particle size decreases throughout the conversion process from Li₂S₂ to Li₂S₆. This suggests that smaller particle sizes facilitate S─S bond formation, thus promoting the transformation from Li₂S₂ to S₈. These theoretical results are consistent with the trends observed in our IPES/UPS experiments, where the d‐band center shifts downward with decreasing particle size (Figure 7d). In our experiments, smaller particle sizes correlate with stronger transformation abilities, with Co@NCNT‐5 demonstrating excellent transformation capability for Li₂S₆ (Figure 7e). It is important to note that Co 3*d* and S 2*p* hybridization occurs during Li insertion and desertion from the NC matrix. Therefore, our theoretical and experimental results confirm that the desertion of Li from the NC matrix facilitates the activation of Li₂S/Li₂S₂, subsequently promoting the transformation to S₈.

**Figure 8:**
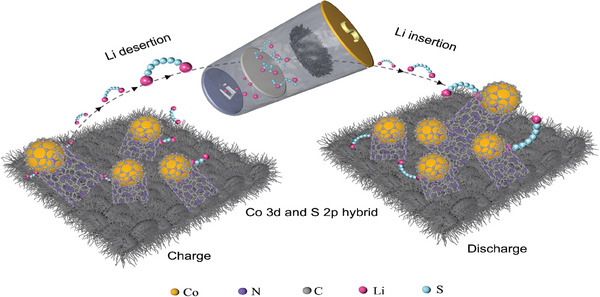
Mechanism diagram.

Based on these findings, we analyzed the mechanism of action in the battery system, as illustrated in **Figure** **8** Besides the absorption role of the Co site, N‐C defects in Co@NCNT‐5 serve as desertion/insertion centers for LiPSs, accompanied by electron transport. Simultaneously, the electrons transfer to adjacent Co 3*d* orbitals, causing their band centers to shift downward and hybridize with S *2p* orbitals. This ultimately increases the covalency of S atoms, enabling S to couple with each other, forming S_8_.

## 3 Conclusion

In summary, this study employed Co@NCNT to modify separators in LSBs. In situ, XRD and Raman spectroscopy, combined with electrochemical data, reveal that during the charging and discharging processes, Li^+^ are inserted and deserted within the (C‐N) matrix. This insertion/extraction process facilitates the pre‐activation of the Li₂S to LiPSs conversion, which is notably dependent on the Co particle size. Once activated, the subsequent conversion of LiPSs to S₈ is significantly promoted. Additionally, we have initiatively introduced IPES/UPS techniques to investigate the influence of particle size and the charging/discharging process on the *d*‐band center of the material. The results indicate that, within the Co@NCNT system, smaller particle sizes correspond to a greater downward shift of the Co 3*d*‐band center. This shift enhances hybridization with the S 2*p* orbitals in the adsorbed LiPSs, improving electron transfer during charging and thus increasing the S covalency. This increased covalency facilitates nucleation and bond breaking in the S redox process, particularly accelerating the conversion between LiPSs to S₈. This study explores new mechanisms in LSBs and provides reliable techniques to support conventional theoretical calculations on the *d*‐band center's impact on charging and discharging mechanisms.

## Conflict of Interest

The authors declare no conflict of interest.

## Supporting information



Supporting Information

## Data Availability

The data that support the findings of this study are available in the supplementary material of this article.
