# Supplementary material for: Direct Observation of Hybridization Between Co 3d and S 2p Electronic Orbits: Moderating Sulfur Covalency to Pre‐Activate Sulfur‐Redox in Lithium–Sulfur Batteries
Source: Adv Sci (Weinh). 2024 Dec 27;12(7):2412038. doi: 10.1002/advs.202412038 (PMC11831454; doi:10.1002/advs.202412038)
Supplement: Supplementary file 1 — Supporting Information [file ADVS-12-2412038-s001.docx]

Supporting Information

**Direct Observation of Hybridization between Co 3*d* and S 2*p*** **Electronic Orbits: Moderating Sulfur Covalency to Pre-Activate Sulfur-Redox in Lithium-Sulfur Batteries**

*Di Wang^//a^, Yaozu Jia^//a^, Qi Jin^a^, Fengying Tian^a^, Qiong Gao^a^,* *Xu Xu^b*^, Huiqing Lu^a^, Lili Wu^a*^, Xinzhi Ma^a*^, and Xitian Zhang^a^*

^a^ *Key Laboratory for Photonic and Electronic Bandgap Materials, Ministry of Education, School of Physics and Electronic Engineering, Harbin Normal University, Heilongjiang 150025, Harbin, China.*

^b^ *State Key Laboratory of Advanced Technology for Materials Synthesis and Processing, School of Materials Science and Engineering, Wuhan University of Technology, Wuhan 430070, Hubei, China.*

**Experimental**

**Materials**

The following materials were used in this study: cobalt nitrate hexahydrate (Co(NO₃)₂·6H₂O), 2-methylimidazole (C₄H₆N₂), hydrophilic CC, anhydrous methanol (CH₃OH), anhydrous ethanol (C₂H₅OH), Ketjen Black (KB), sublimed sulfur (S) from Beijing Dekodo Gold, conductive carbon black (Super P), N-methyl-2-pyrrolidone (NMP) dispersant, polyvinyl phenol adhesive (5% PVDF), Li-S battery electrolyte, and polypropylene separator. All chemicals, including anhydrous ethanol, were of analytical grade and did not require further purification. Ultrapure water (18.25 MΩ cm) was obtained using a UPT-II purifier system and was used for all experiments.

The following equipment was employed: magnetic stirrer, constant temperature water bath, ultrasonic cleaning instrument, constant temperature drying oven, electronic balance, tube furnace, reaction kettle, mortar, pipette gun, coating machine, vacuum drying oven, tablet press, high-precision electronic balance, glove box, small hydraulic button battery sealing machine, scanning electron microscope (Hitachi, SU70), battery test system (LAND, CT3001A), electrochemical workstation (Biologic, VMP3), and X-ray diffractometer.

**Synthesis of Co@NCNT-CC Interlayers**

A fixed ratio of cobalt nitrate hexahydrate (Co(NO₃)₂·6H₂O) and 2-methylimidazole (C₄H₆N₂) was precisely weighed using an electronic analytical balance and then dissolved separately in pre-measured 100 mL portions of anhydrous methanol. The resulting solutions were stirred with a magnetic stirrer at 500 rpm for 5 minutes. CC pieces of appropriate size were immersed in a 2-methylimidazole solution in ethanol and subjected to ultrasonic treatment for 15 minutes. The two solutions were then combined and placed in a constant temperature water bath for 10 minutes. The mixture was vigorously stirred at 1000 rpm for 10 minutes, followed by slow stirring for 12 hours to ensure complete reaction. The obtained sample was washed with anhydrous ethanol and dried in an oven at 60°C for 12 hours to yield the ZIF-67@CC material.

The dried material was cut to the desired size, placed into a porcelain boat, and annealed in a tube furnace at 435°C under a flow of hydrogen and argon gas for 4.5 hours. After cooling to room temperature, the material was cut into circular sheets with a diameter of 13 mm using a tablet press, resulting in Co@NCNT-CC interlayers suitable for button battery assembly.

In our case, the areal loading of CC and Co@NCNT are calculated to be 1.7 mg cm^-1^ based on the function that (Mass_Co@NCNT-CC_-Mass_CC_)/Surface area, where the surface area is calculated to be 1.32 cm^-2^ base on the 13 mm diameter of separator and 0.01997 and 0.02219 g for Mass_Co@NCNT-CC_ and Mass_CC_, respectively (Figure S1).

**Preparation of S/KB Composites and Positive Electrode Fabrication**

S/KB composites were prepared using the hot melt diffusion method. Sublimed S and KB were mixed and ground in a mortar at a mass ratio of 7:3. The thoroughly ground mixture was transferred to a Teflon-lined reactor and heated to 155°C, maintaining this temperature for 12 hours. After cooling, 1/8 of the product’s weight of conductive carbon black was added and the mixture was ground again in a mortar.

The positive electrode was formulated with 70 wt% S/KB by mixing 80 wt% of the active material, 10 wt% conductive carbon black, and 10 wt% PVDF in NMP to create a slurry. The slurry was ultrasonicated for 1 hour to ensure uniform dispersion. The resulting paste was coated onto aluminum foil using a coater, dried in a vacuum oven at 60°C for 12 hours, and cut into 13 mm round sheets using a tablet press for assembly into button batteries. The S loading was maintained at 1.0 mg cm⁻².

**Battery Assembly**

The battery assembly was performed in a glove box filled with pure argon gas (H₂O < 0.1 ppm, O₂ < 0.1 ppm) using CR2032 battery cases. The negative electrode was a lithium plate with a diameter of 15.6 mm, and the separator was a polypropylene film with a diameter of 19.0 mm.

To assemble the battery, a droplet of Li-S battery electrolyte was carefully placed at the center of the positive electrode shell. The positive electrode was then positioned onto the positive surface and covered with a Co@NCNT-CC middle layer. Subsequently, 80 μL of LSBs electrolyte was added onto the middle layer. The separator was inserted from the side to ensure complete saturation. Following this, the lithium sheet, gasket, shrapnel, and negative shell were sequentially added. The assembled battery was then placed into a battery sealing machine and pressed into button battery format.

**Electrochemical Testing**

*Full Cell CV Measurement*

For the measurement of full cell CV, the materials used included CR2032 positive and negative cases, LSBs electrolyte, blank diaphragm, CS positive tabs, and Co@NCNT-CC. These materials were assembled into a lithium-sulfur full cell incorporating the Co@NCNT-CC material. The cell was allowed to stand for two hours before testing.

CV curves and AC impedance spectra of the Co@NCNT-CC cell were measured using an electrochemical workstation. The measurements were conducted within a voltage window of 1.8 to 2.8 V (vs. Li^+^/Li) and at scanning rates of 0.1 to 0.5 mV s⁻¹. The results demonstrated that the Co@NCNT-CC cell exhibits excellent performance as an LSBs.

*Battery Performance Testing*

The constant current charge/discharge curves of the batteries were measured at various rates, including 0.1, 0.2, 0.5, 1, 2, 5, and 10 C, within the voltage range of 1.8 to 2.8 V (vs. Li^+^/Li), using the LAND CT3001A constant current charge/discharge system.

*Preparation of Electrode Ink and Cell Assembly.*

For the preparation of the electrode ink, 20 mg of Co@NCNT and 44.4 mg of 5% PVDF were added to 200 μL of NMP solution and sonicated for 1 hour. The resulting ink was then applied to hydrophobic carbon paper and dried for 12 hours.

For cell assembly, the following sequence was used: the positive shell was placed first, with the carbon paper material facing up. Then, 20 μL of 0.1 mol L^-1^ Li₂S₆ electrolyte was added, followed by a blank separator. The carbon paper material was placed facing down, and the assembly was completed with shrapnel, spacer, and negative shell arranged from bottom to top. The cells were allowed to rest for two hours before a final pressing was performed, maintaining the sequence of positive shell, carbon paper material facing up, and negative shell. CV curves and AC impedance spectra of the symmetric cells were subsequently measured using an electrochemical workstation.

*Li_2_S Deposition Tests*

10 mg of Co@NCNT was added to 150 µL of anhydrous ethanol and sonicated for 1 hour. For cell assembly, the following sequence was used: the positive shell was placed first with the carbon paper material facing up, followed by 20 μL of Li₂S₈ electrolyte, a blank separator, and the carbon paper material facing down. The assembly was completed with shrapnel, spacer, and negative shell arranged from bottom to top. The cells were allowed to rest for two hours before a final pressing, which was performed for 30 minutes with the same sequence of positive shell, carbon paper material facing up, and negative shell. Li₂S deposition was evaluated using an electrochemical workstation.

*DFT*

All spin-polarized density-functional theory (DFT) computations were performed using the Vienna ab initio simulation package (VASP)^[^^[[1]](#endnote-1)]^ based on the projector augmented wave (PAW)^[^^[[2]](#endnote-2)]^ method. Electron-ion interactions were described using standard PAW potentials. A plane-wave basis set was employed to expand the smooth part of the wave functions with a cutoff kinetic energy of 400 eV. For the Electron-electron exchange and correlation interactions, the functional parametrized by Perdew-Burke-Ernzerhof (PBE)^[^^[[3]](#endnote-3)]^, a form of the general gradient approximation (GGA), was used throughout. The Van der Waals interaction was described via the DFT-D3BJ method^[^^[[4]](#endnote-4)]^.

To study the mechanistic chemistry of surface reactions, the surface was modelled with a slab model. A suffciently large vacuum region of 15 Å was used to ensure the periodic images were well separated. During the geometry optimizations, the bottom atoms were fixed at the bulk position when the surface properties were calculated. In this work, the Brillouin-zone integrations were conducted using Monkhorst-Pack grids^[^^[[5]](#endnote-5)]^ of special points with a separation of 0.04 Å^-1^. The convergence criterion for the electronic self-consistent loop was set to 10^-5^ eV. The atomic structures were optimized until the residual forces were below 0.03 eV Å^-1^.


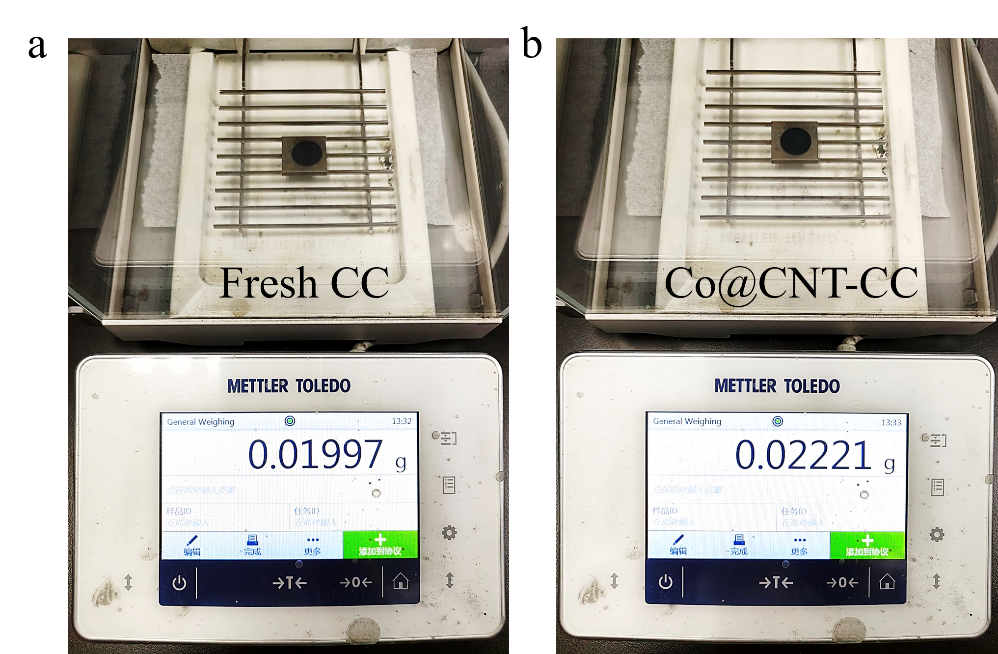


Figure S1. The weight digitals of CC and Co@NCNT-CC, respectively.


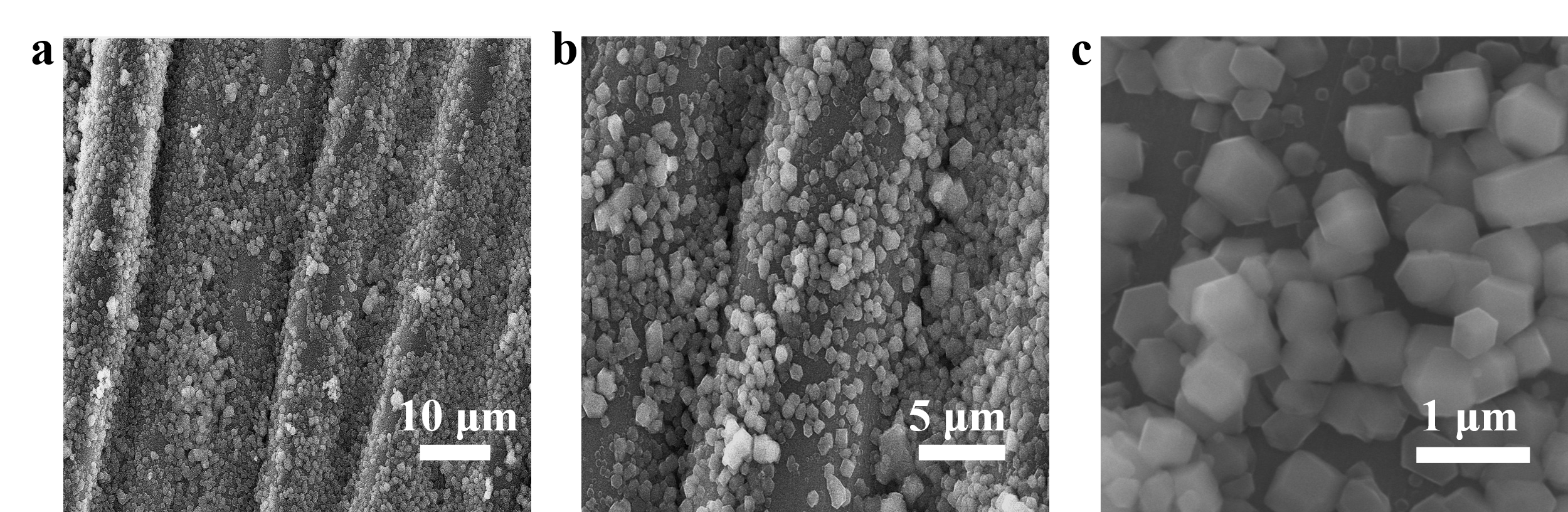


**Figure S2.** (a-c) SEM images of ZIF-67@CC, respectively.


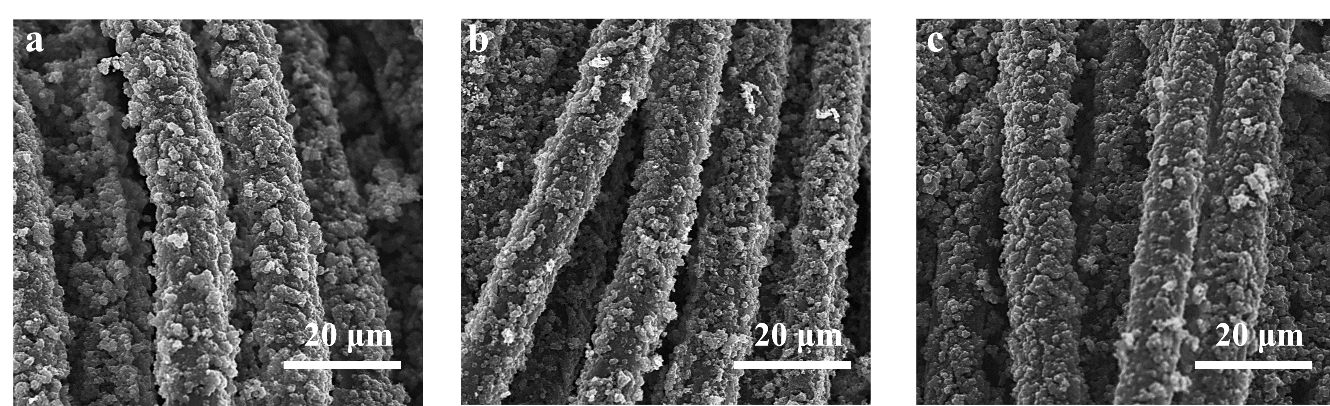


**Figure S3.** (a-c) SEM images of Co@NCNT-5, Co@NCNT-10, Co@NCNT-20, respectively.


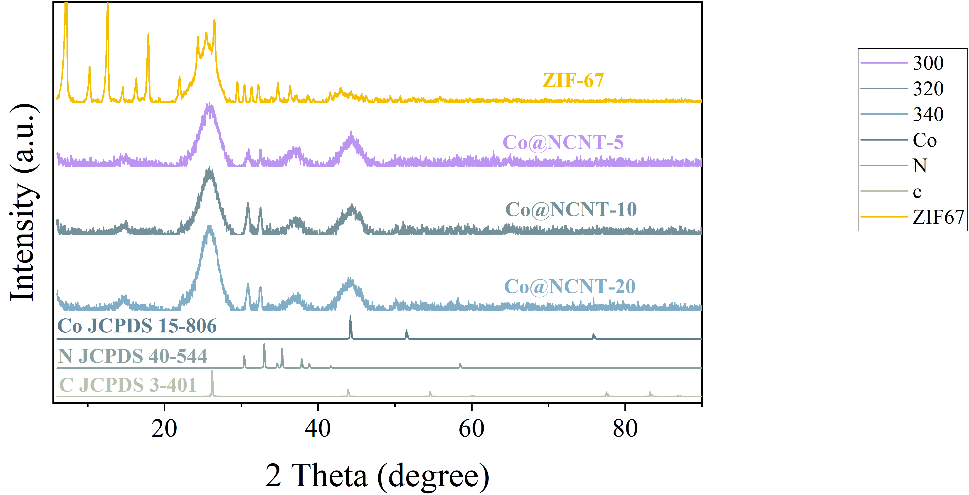


**Figure S4.** XRD patterns of ZIF-67, Co@NCNT-5, Co@NCNT-10, Co@NCNT-20, respectively.


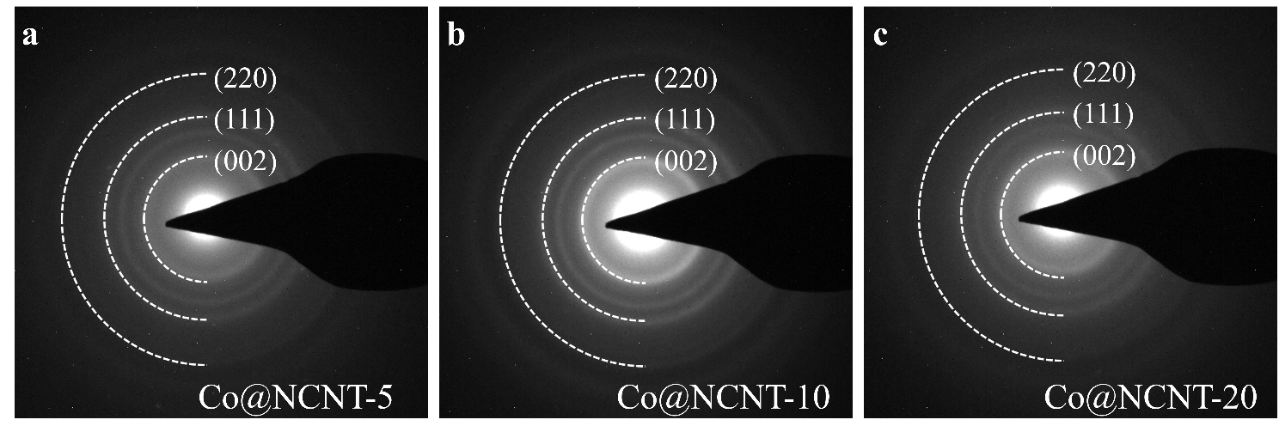


**Figure S5.** Selected electron diffraction patterns of Co@NCNT-5, Co@NCNT-10, Co@NCNT-20, respectively.


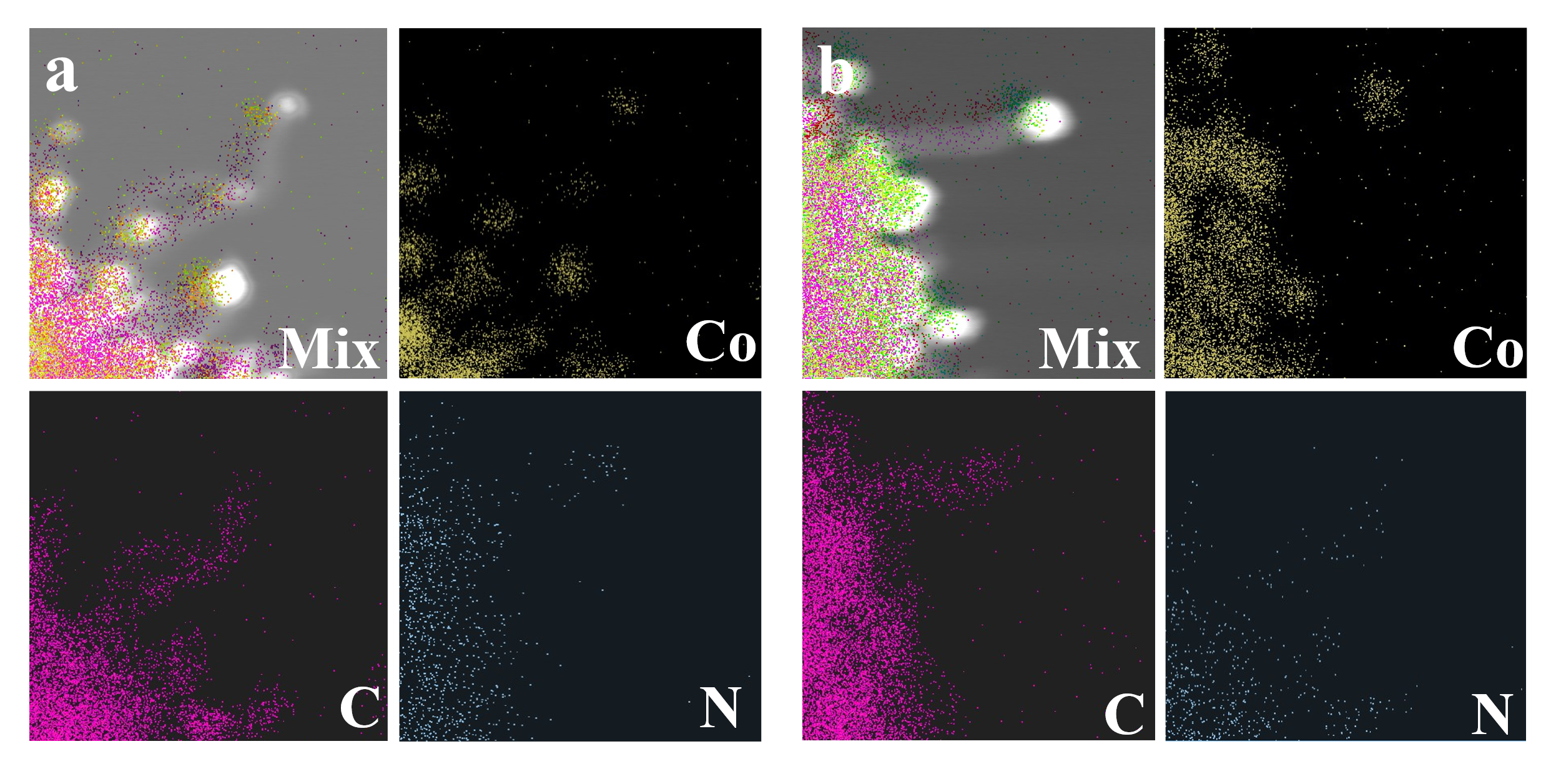


**Figure S6.** (a, b) Elemental mappings of Co@NCNT-10, Co@NCNT-20, respectively.


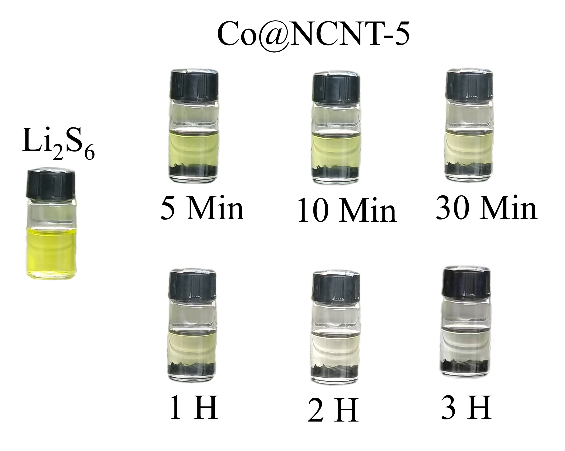


**Figure S7.** Li_2_S_6_ adsorption of Co@NCNT-5.


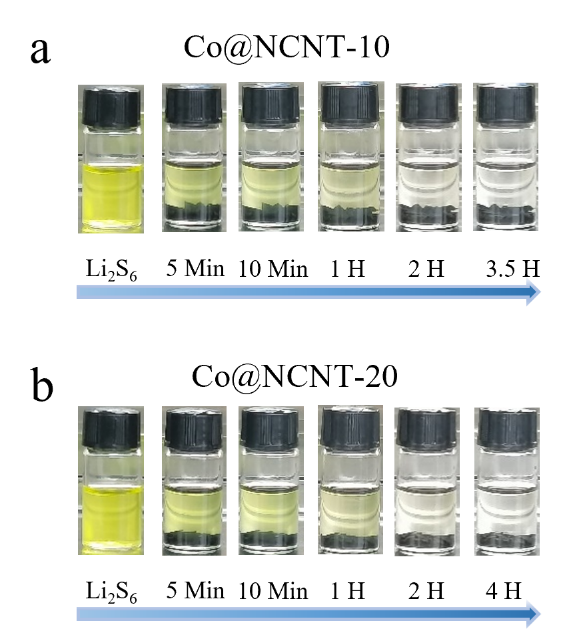


**Figure S8.** (a, b) Li_2_S_6_ adsorption of Co@NCNT-10 and Co@NCNT-20.


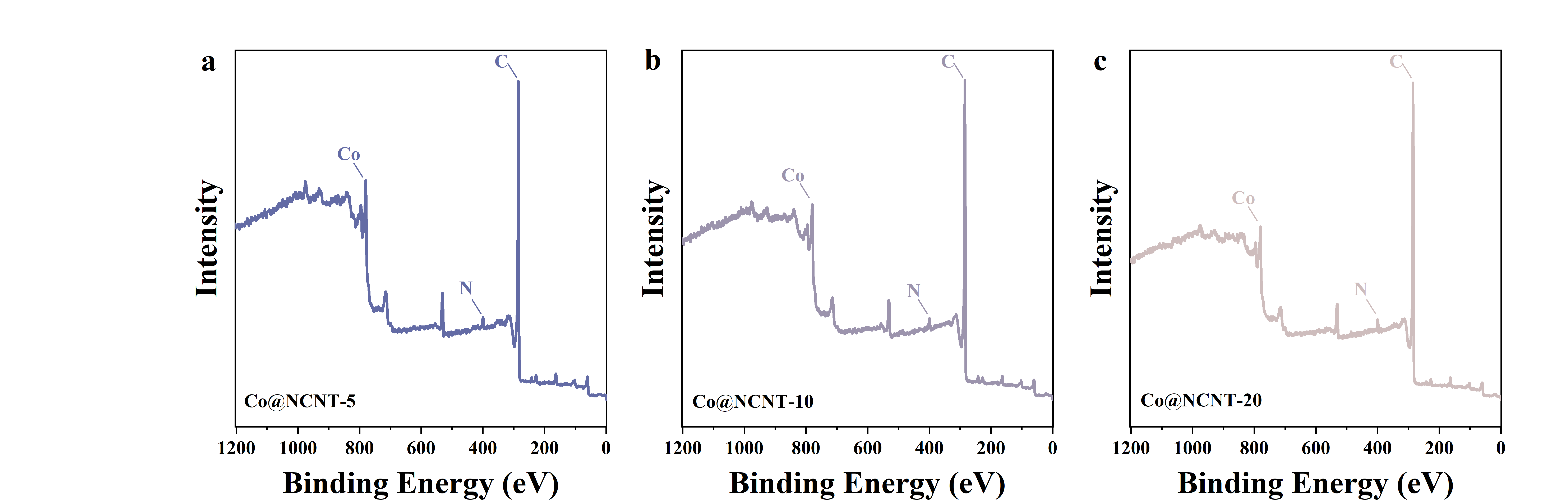


**Figure S9.** Survey XPS spectra. (a) Co@NCNT-5, (b) Co@NCNT-10, and (c) Co@NCNT-20.


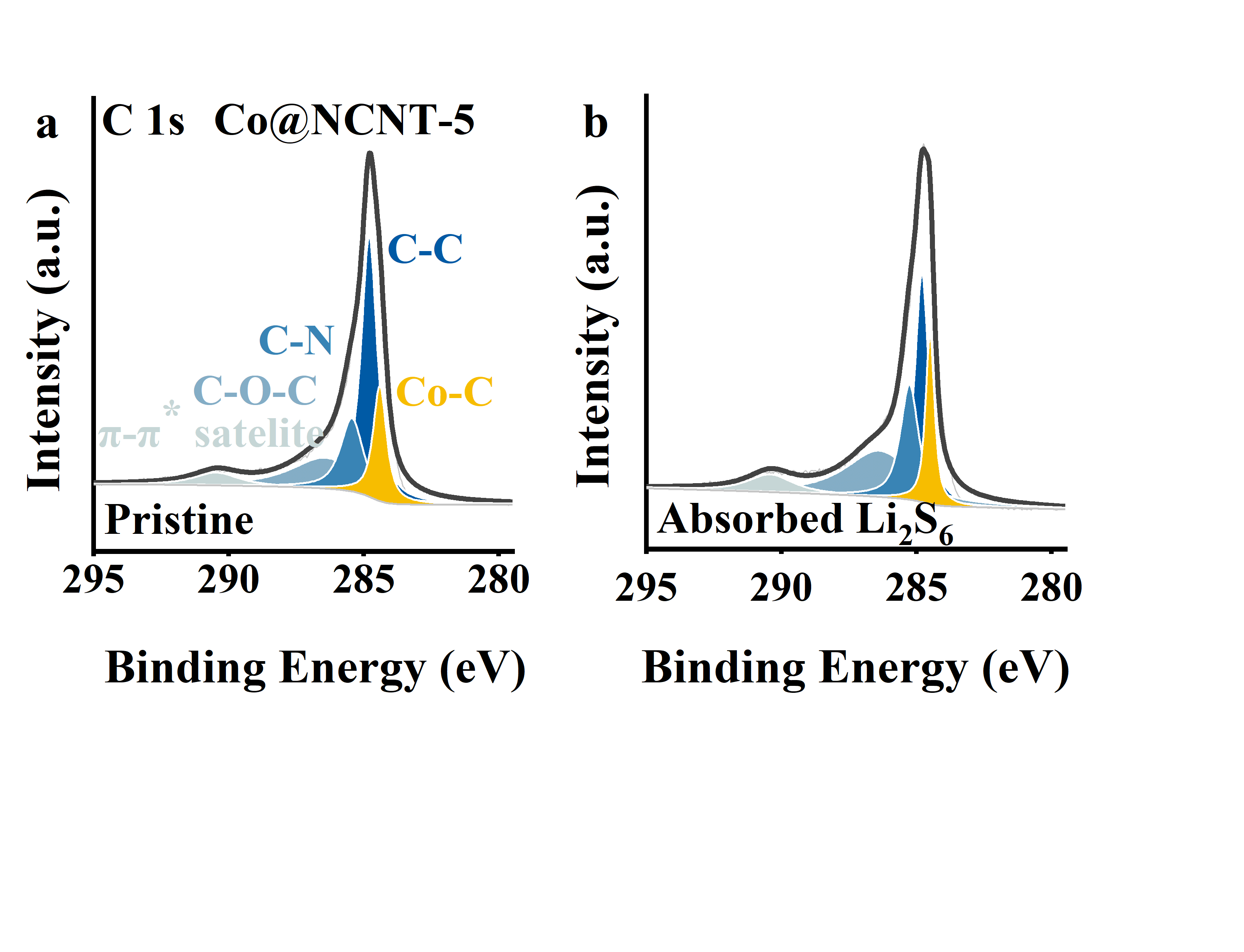


**Figure S10.** High-resolution C 1*s* XPS spectra of Co@NCNT-5.


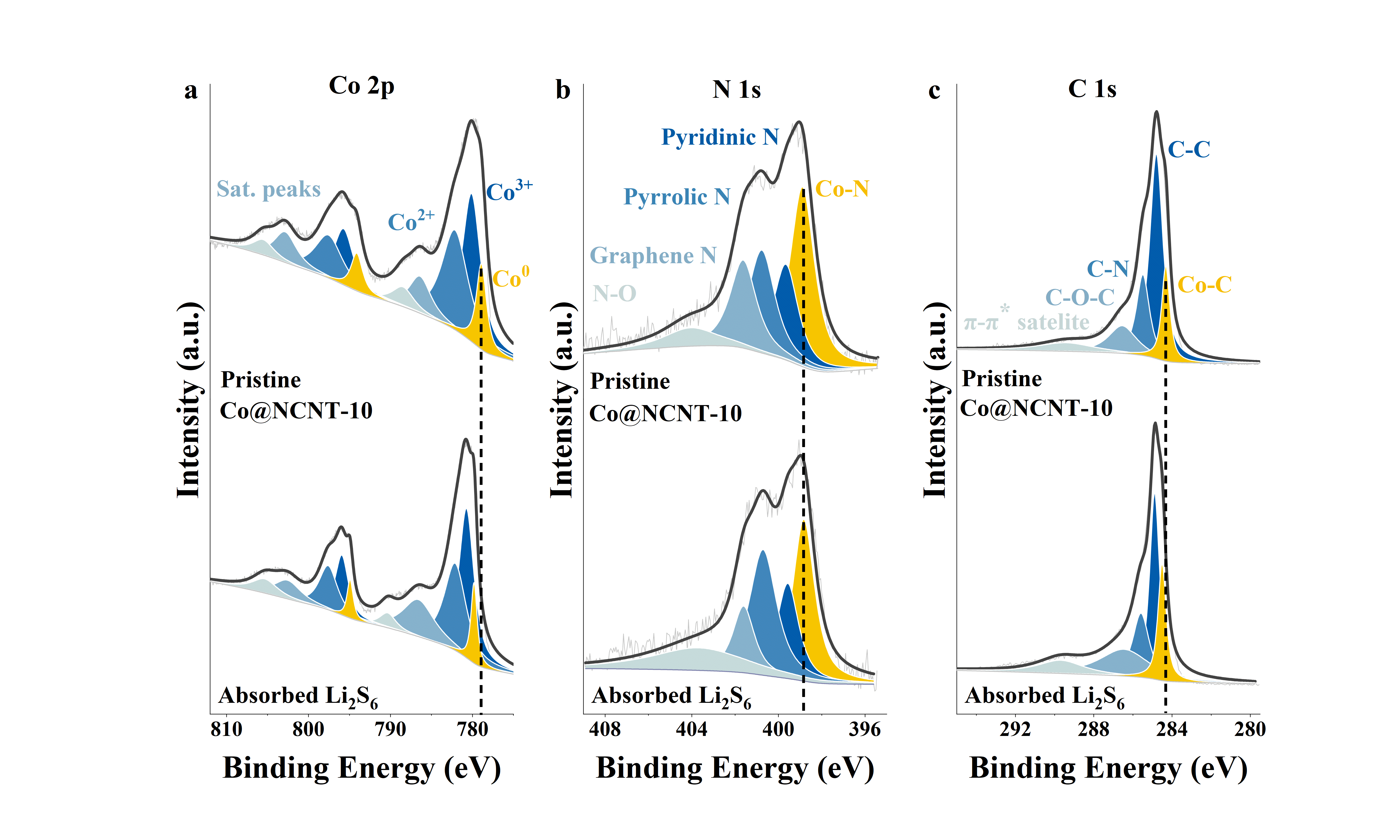


**Figure S11.** High-resolution XPS spectra of Co@NCNT-10, (a) Co 2p, (b) N 1s, (c) C 1s.


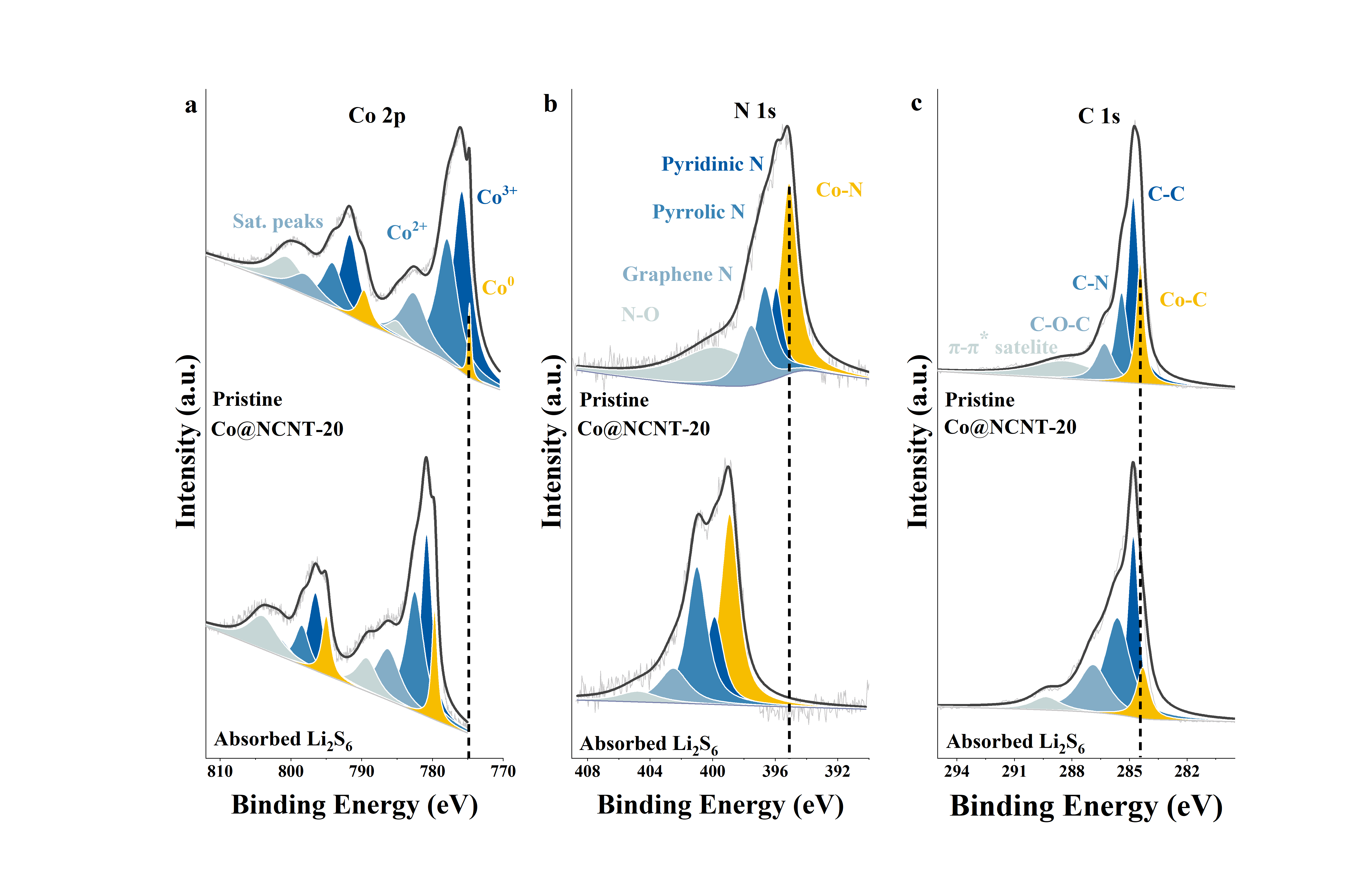


**Figure S12.** High-resolution XPS spectra of Co@NCNT-20, (c) Co 2p, (d) N 1s, (e) C 1s.


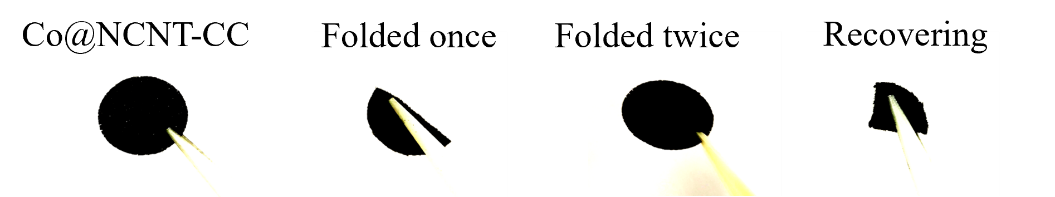


**Figure S13.** Bending tests on Co@NCNT-5.


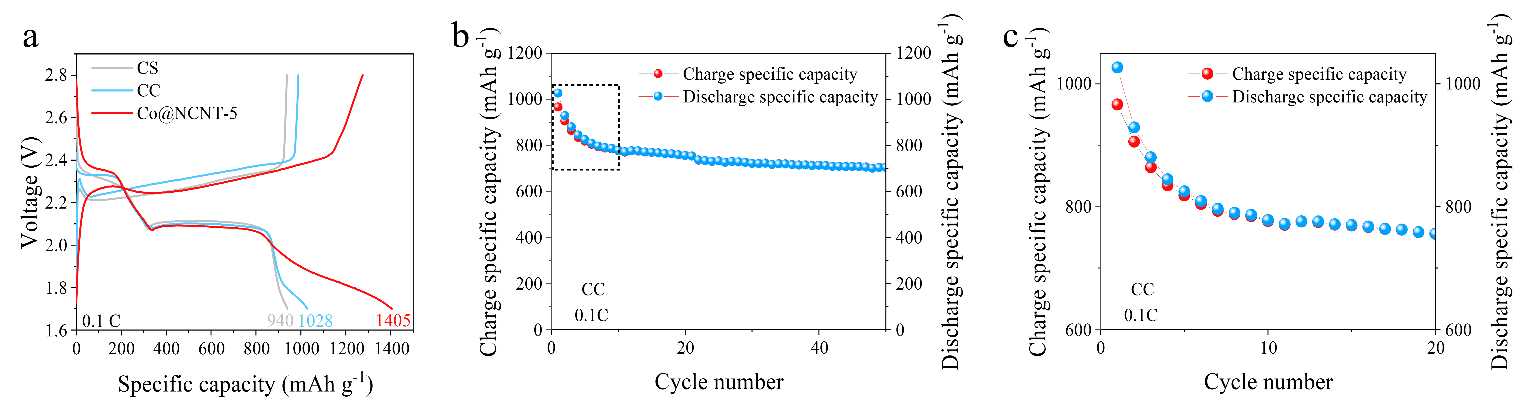


**Figure S14.** (a) GCD curves of CS-LSB, CC-LSB and Co@NCNT-5-LSB; (b, c) Cycle diagram at 0.1 C magnification of CC-LSB.


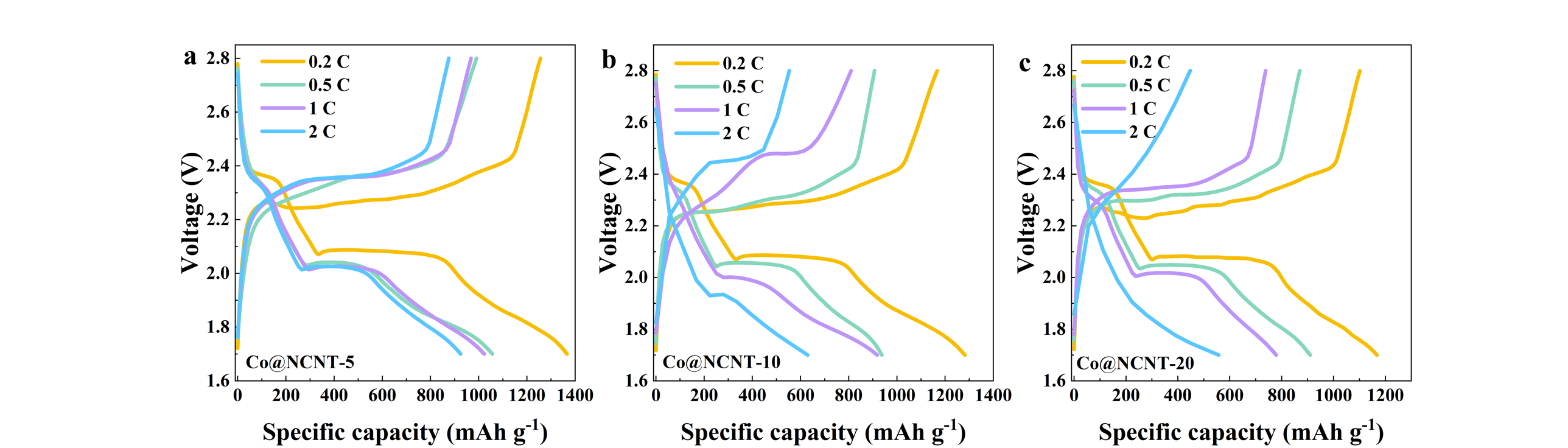


**Figure S15.** Rate dependences of charge-discharge curves of Co@NCNT-5, 10, 20.


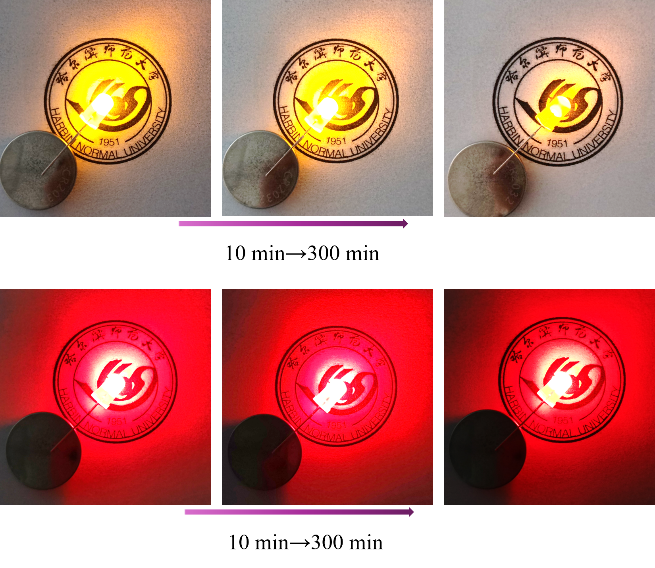


**Figure S16.** Battery luminous duration chart.


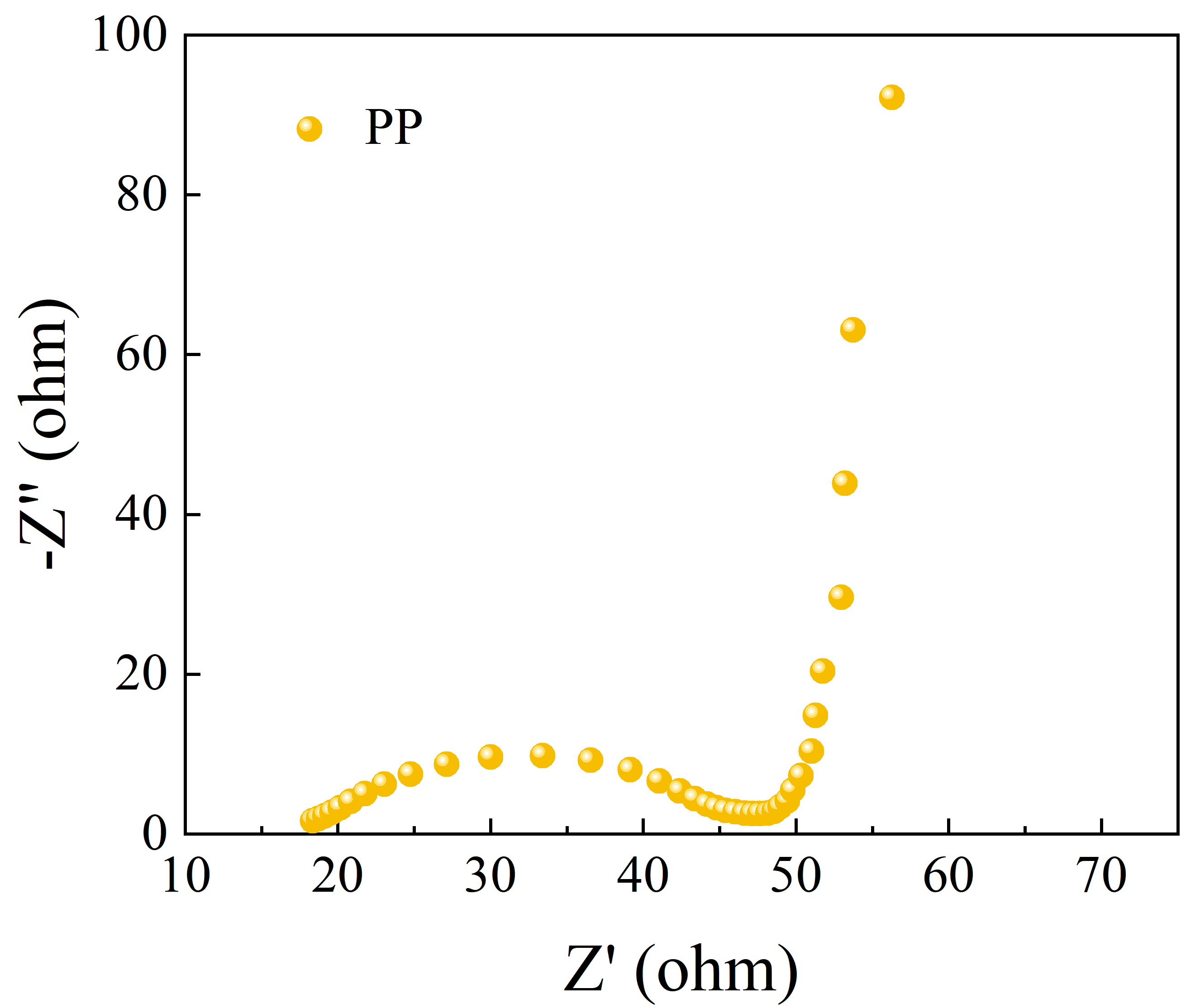


**Figure S17.** Nyquist plots of PP-coupled LSB.


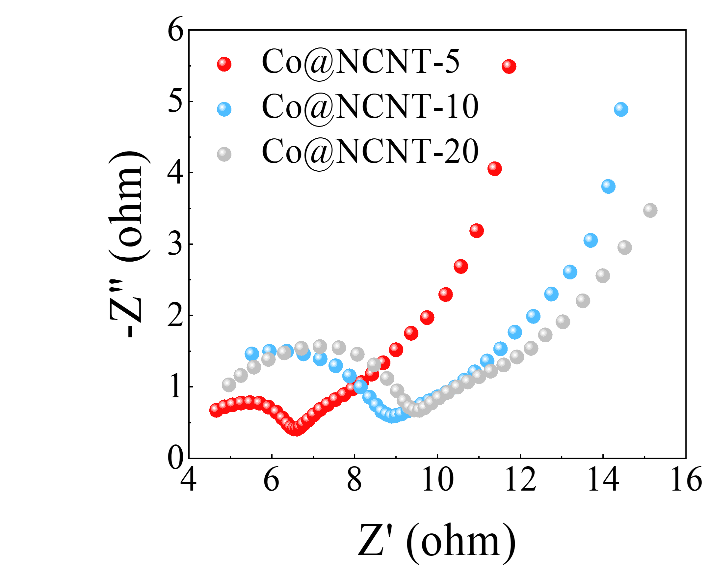


**Figure S18.** Nyquist plots of Co@NCNT-5, 10, 20 coupled LSBs.


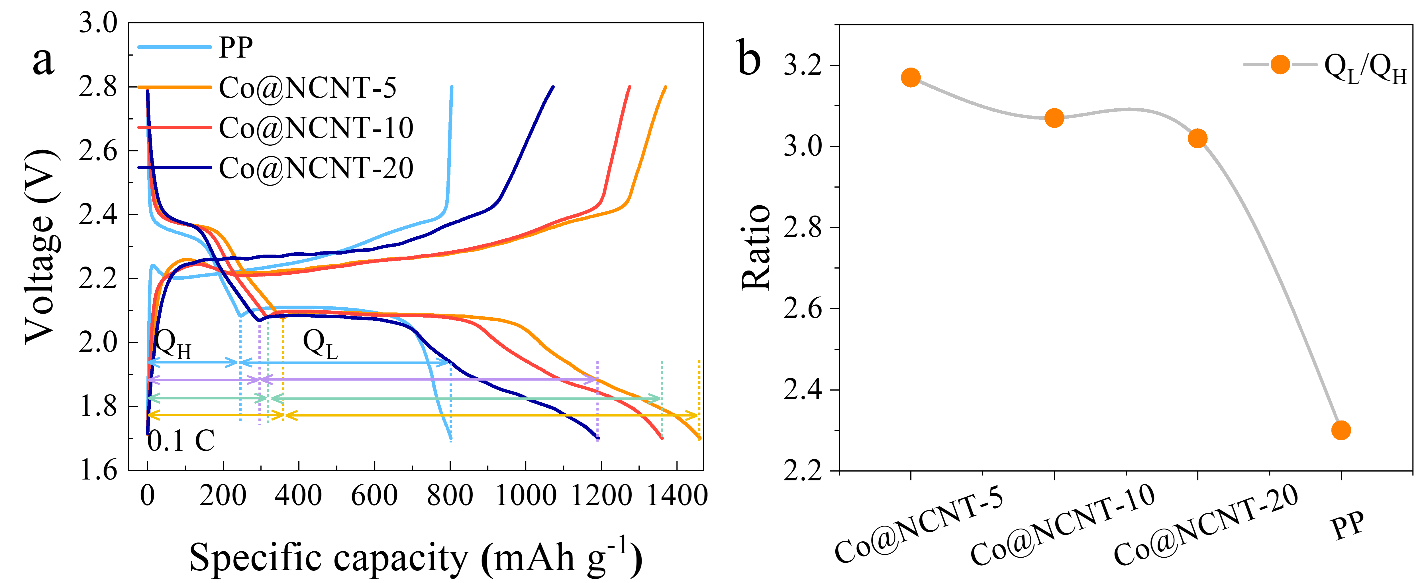


**Figure S19.** (a) GCD curves, (b) Q_L_/Q_H_ plots.


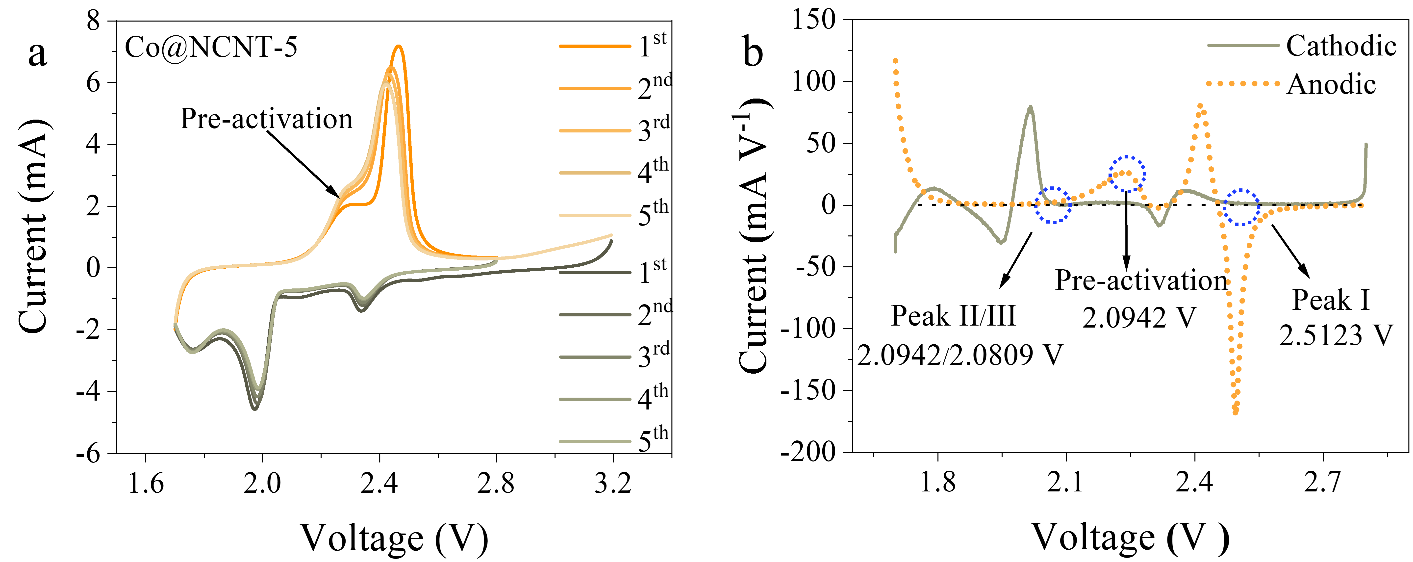


**Figure S20.** (a, b) CV curves and the corresponding differential plots.

**
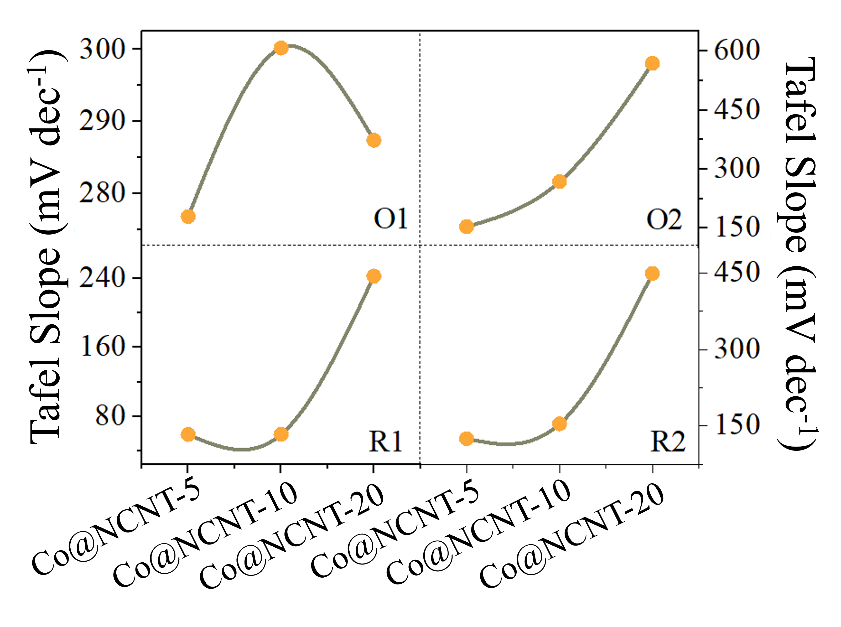
**

**Figure S21.** Tafel slopes of O_1_, O_2_, R_1_, R_2_.

**
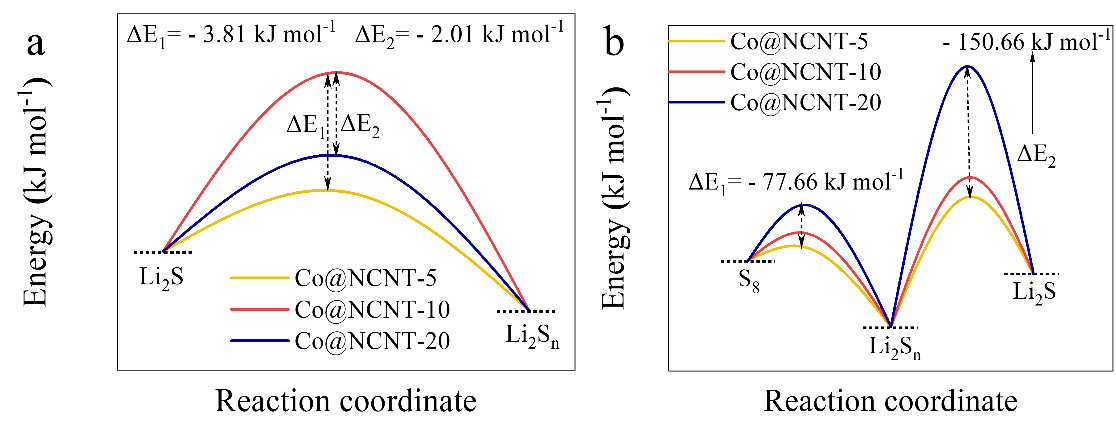
**

**Figure S22.** (a, b) Activation energy curves.


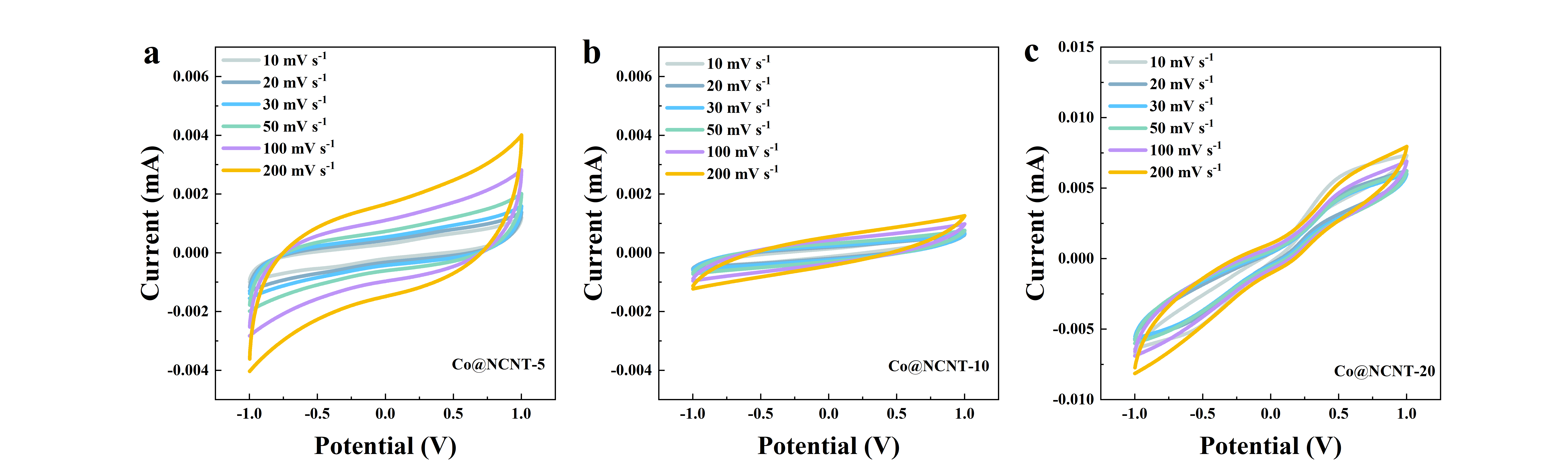


**Figure S23.** CV curves of Co@NCNT-5, 10, 20 symmetric cells.


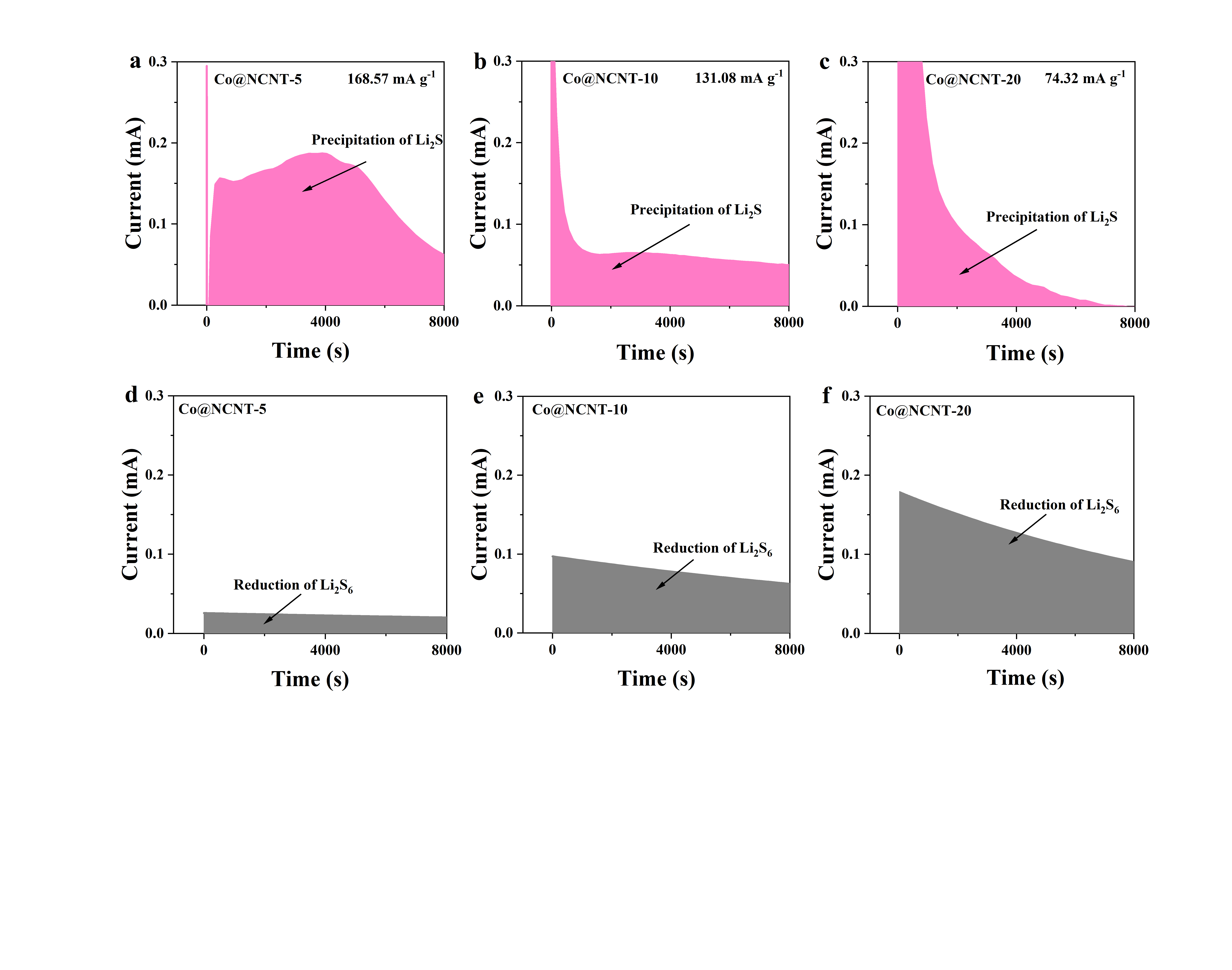


**Figure S24.** Li_2_S deposition tests.


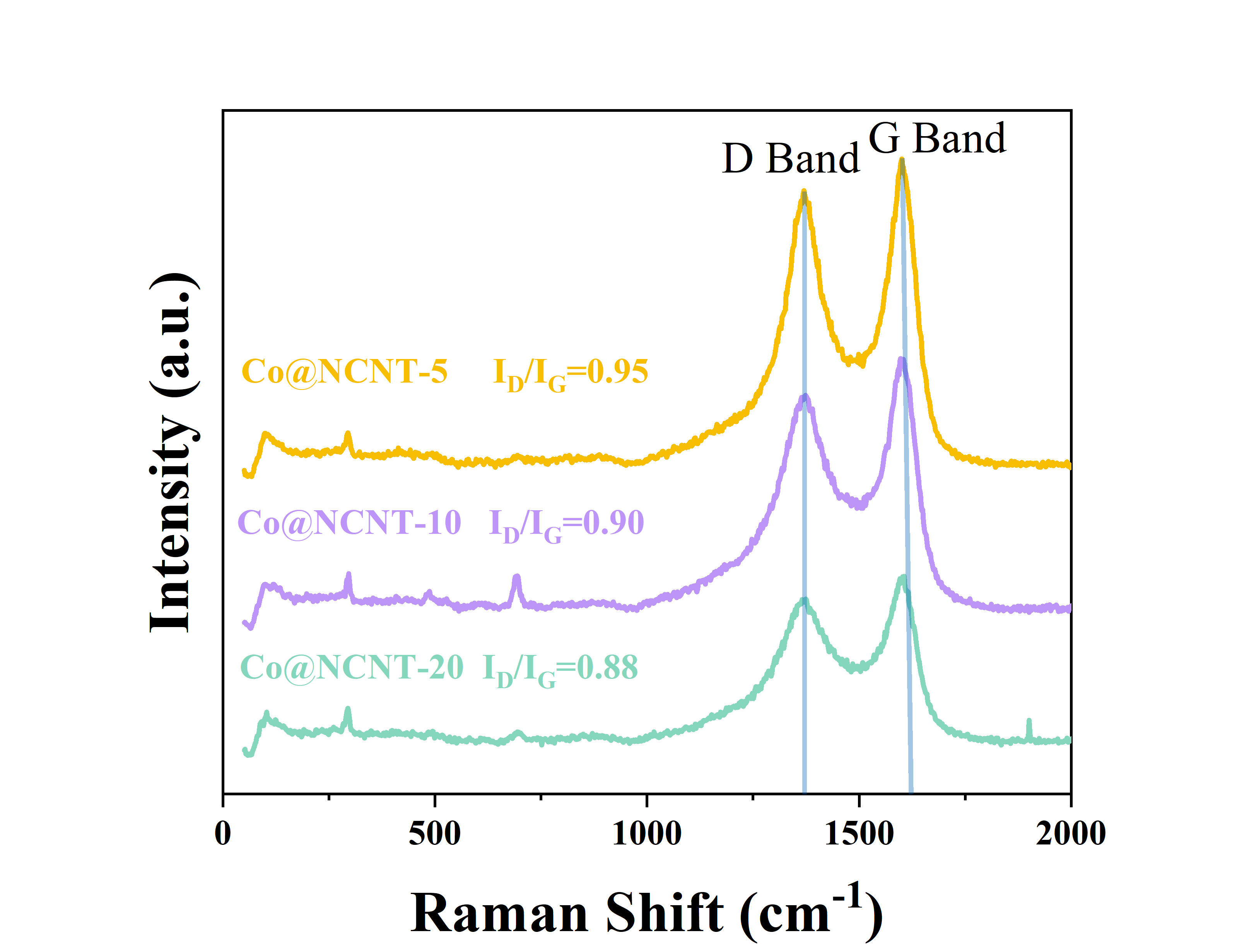


**Figure S25.** Raman spectra of Co@NCNT-5, Co@NCNT-10, and Co@NCNT-20.


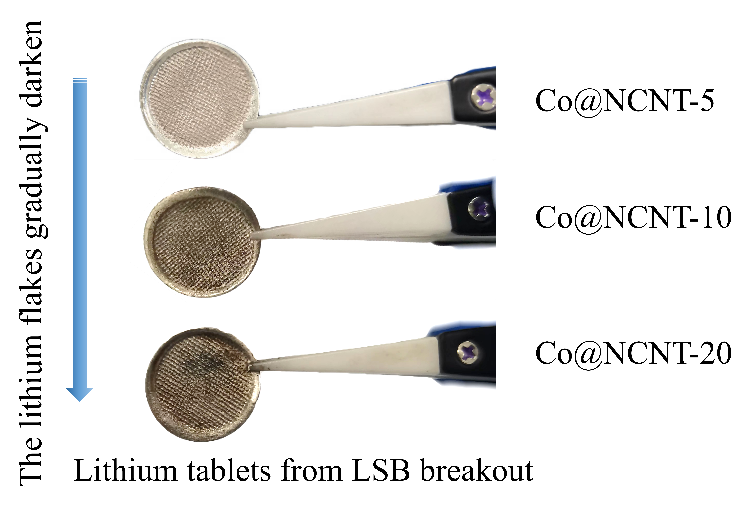


**Figure S26.** Li anodes of LSBs post discharging/charging.


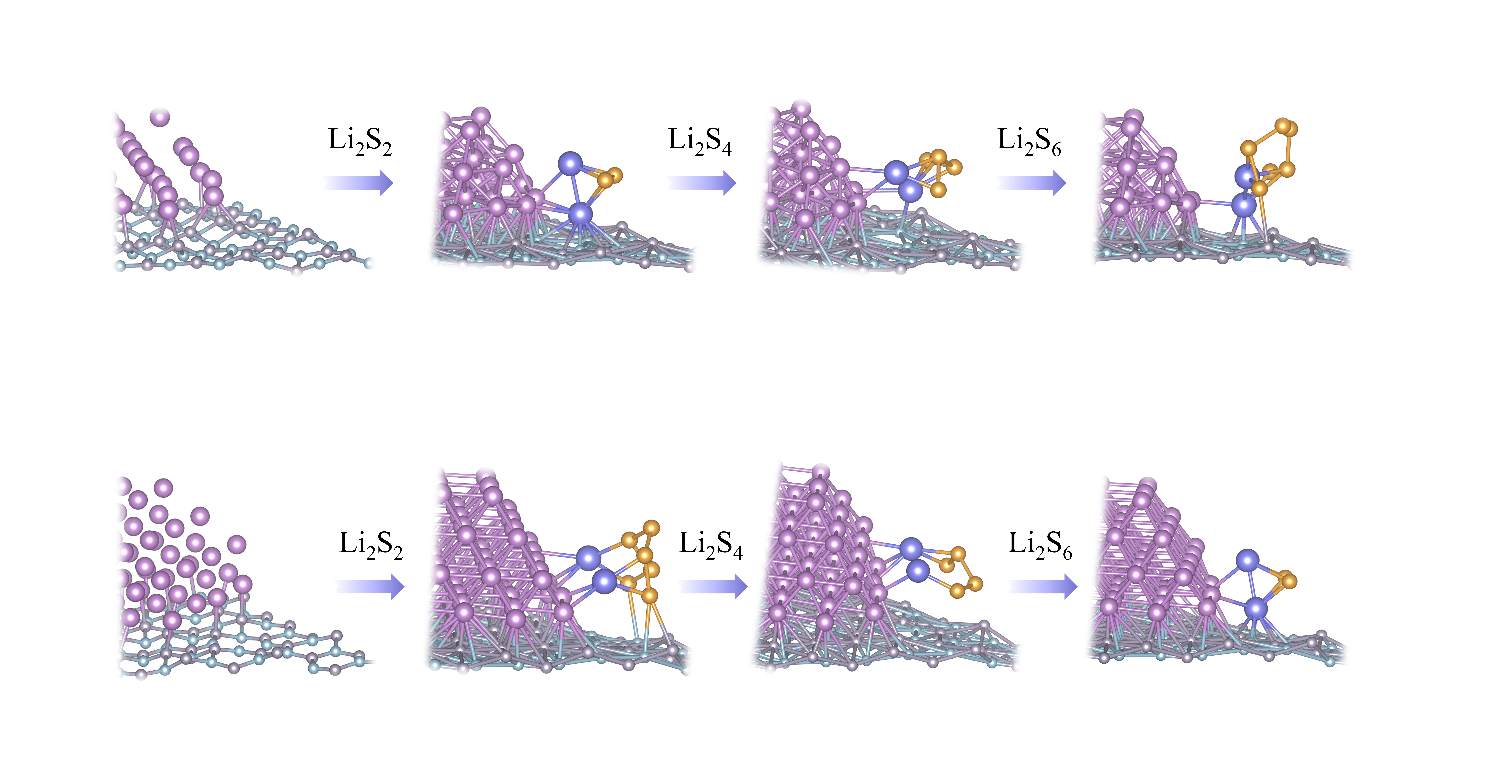


**Figure S27.** Co@NCNT-10 and Li_2_S_2_,Li_2_S_4_ and Li_2_S_6_ interact with Co@NCNT-10, respectively.


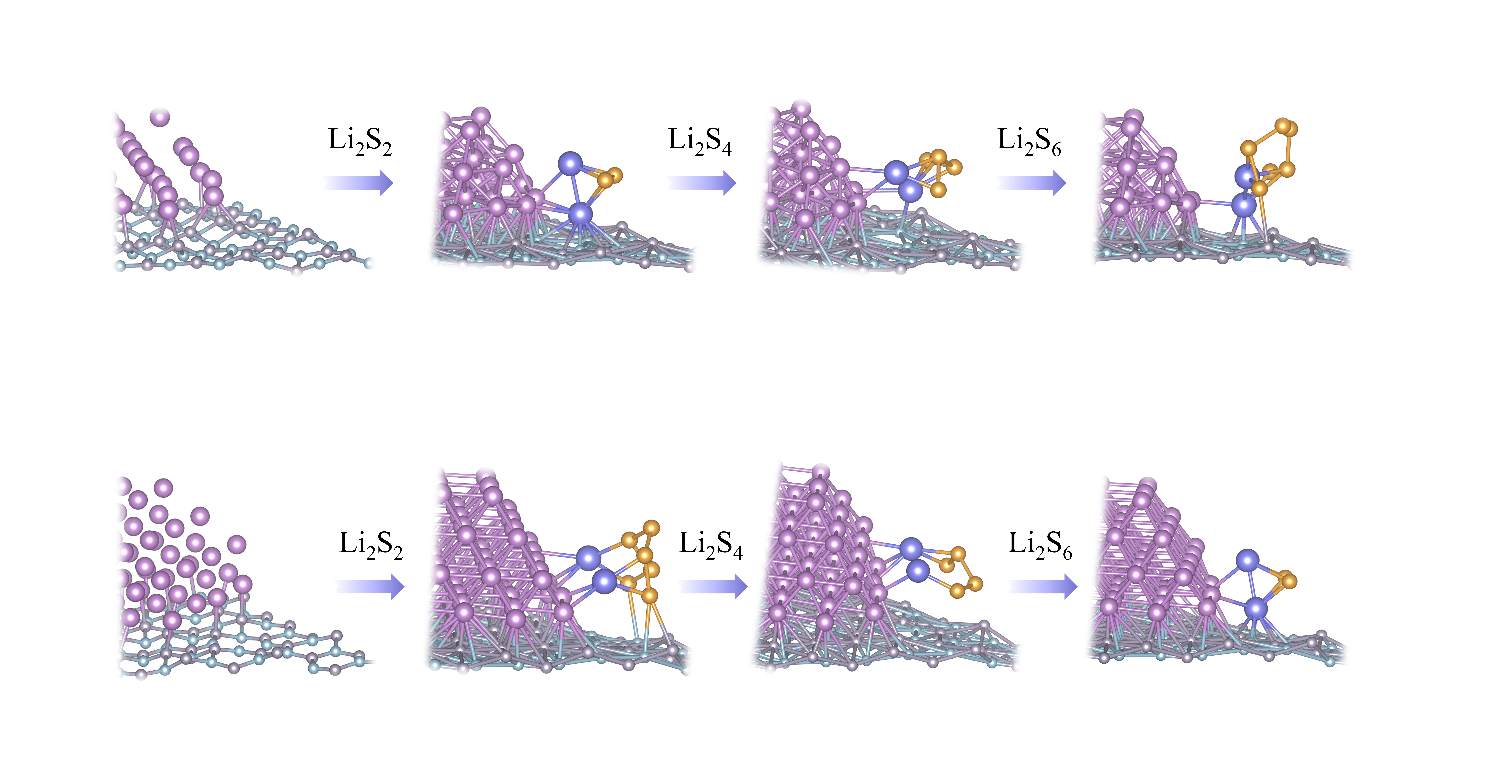


**Figure S28.** Co@NCNT-20 and Li_2_S_2_,Li_2_S_4_ and Li_2_S_6_ interact with Co@NCNT-20, respectively.

**Table S1.** Percentages of individual Co-related substances calculated from high-resolution Co 2*p* spectra of Co@NCNT-5.

| **Sample** | **Spin-orbit coupling quantum number** | **Peak name** | **Percentage value** |
| --- | --- | --- | --- |
| **Co@NCNT-5** | Co 2p_3/2_ | Co^0^ | 4.26% |
|  |  | Co^3+^ | 20.88% |
|  |  | Co^2+^ | 26.42% |
|  |  | Sat. peaks | 17.04% |
|  | Co 2p_1/2_ | Co^0^ | 3.41% |
|  |  | Co^3+^ | 7.67% |
|  |  | Co^2+^ | 10.01% |
|  |  | Sat. peaks | 10.31% |

**Table S2.** Percentages of individual Co-related substances calculated from high-resolution Co 2*p* spectra of Co@NCNT-5 post-Li_2_S_6_ absorption.

| **Sample** | **Spin-orbit coupling quantum number** | **Peak name** | **Percentage value** |
| --- | --- | --- | --- |
| **Co@NCNT-5** | Co 2p_3/2_ | Co^0^ | 6.27% |
|  |  | Co^3+^ | 15.28% |
|  |  | Co^2+^ | 27.43% |
|  |  | Sat. peaks | 18.62% |
|  | Co 2p_1/2_ | Co^0^ | 2.59% |
|  |  | Co^3+^ | 5.09% |
|  |  | Co^2+^ | 13.32% |
|  |  | Sat. peaks | 11.4% |

**Table S3.** Percentages of individual N-related substances calculated from high-resolution N 1s spectra of Co@NCNT-5 post-Li_2_S_6_ absorption.

| **Sample** | **Peak name** | **Percentage value** |
| --- | --- | --- |
|  | Metal-N | 21.28% |
|  | Pyridinic N | 21.20% |
| **Co@NCNT-5** | Pyrrolic N | 33.85% |
|  | Graphene N | 14.18% |
|  | N-O | 9.49% |

**Table S4.** Percentages of individual N-related substances calculated from high-resolution N 1*s* spectra of Co@NCNT-5 post-Li_2_S_6_ absorption.

| **Sample** | **Peak name** | **Percentage value** |
| --- | --- | --- |
|  | Metal-N | 29.10% |
|  | Pyridinic N | 19.55% |
| **Co@NCNT-5** | Pyrrolic N | 20.32% |
|  | Graphene N | 16.28% |
|  | N-O | 14.75% |

**Table S5.** Percentages of individual N-related substances calculated from high-resolution N 1*s* spectra of Co@NCNT-5 post-charging.

| **Sample** | **Peak name** | **Percentage value** |
| --- | --- | --- |
|  | Metal-N | 21.23% |
|  | Pyridinic N | 23.63% |
| **Co@NCNT-5** | Pyrrolic N | 29.78% |
|  | Graphene N | 11.14% |
|  | N-O | 14.22% |

**Table S6.** Percentages of individual N-related substances calculated from high-resolution N 1*s* spectra of Co@NCNT-5 post-discharging.

| **Sample** | **Peak name** | **Percentage value** |
| --- | --- | --- |
|  | Metal-N | 11.53% |
|  | Pyridinic N | 35.11% |
| **Co@NCNT-5** | Pyrrolic N | 19.26% |
|  | Graphene N | 14.64% |
|  | N-O | 19.46% |

**Table S7.** Percentages of individual C-related substances calculated from high-resolution C 1*s* spectra of Co@NCNT-5.

| **Sample** | **Peak name** | **Percentage value** |
| --- | --- | --- |
| **Co@NCNT-5** | Co-C | 15.63% |
|  | C-C | 41.19% |
|  | C-N | 16.55% |
|  | C-O-C | 20.83% |
|  | π-π* satellite | 5.8% |

**Table S8.** Percentages of individual C-related substances calculated from high-resolution C 1*s* spectra of Co@NCNT-5 post-Li_2_S_6_ absorption.

| **Sample** | **Peak name** | **Percentage value** |
| --- | --- | --- |
| **Co@NCNT-5** | Co-C | 14.50% |
|  | C-C | 29.15% |
|  | C-N | 19.54% |
|  | C-O-C | 29.32% |
|  | π-π* satellite | 7.49% |

**Table S9.** Performance comparison.

|  | Samples | Rate (C) | Capacity decay (% per cycle) | References |
| --- | --- | --- | --- | --- |
| 1 | IPHHCS | 10 | 0.1200% | [28] |
| 2 | CNB-TiC@CFN/S | 10 | 0.1042% | [29] |
| 3 | CNB-TiC@CNF | 10 | 0.1042% | [30] |
| 4 | Ni/Ni_3_N | 5 | 0.1000% | [31] |
| 5 | SKB | 5 | 0.0900% | [32] |
| 6 | V-VNC | 5 | 0.087% | [33] |
| 7 | CoS_2_-MgS | 5 | 0.0800% | [34] |
| 8 | Co_3-x_Mn_x_O_4_ | 10 | 0.0770% | [35] |
| 9 | DC/AO-CoVSe | 5 | 0.0660% | [36] |
| 10 | 12nm-ZnO | 10 | 0.0633% | [37] |
| 11 | CoB/NCPS | 5 | 0.0600% | [38] |
| 12 | BNNs@CNFs | 5 | 0.0460% | [39] |
| 13 | PW_12_O_40_ | 10 | 0.041% | [40] |
| 14 | MnO_2_/CN | 5 | 0.0400% | [41] |
| 15 | Co_3_O_4_/ZnO | 2 | 0.0390% | [42] |
| 16 | CoS_2_ | 2 | 0.0340% | [43] |
| 17 | S-DIB | 10 | 0.0300% | [44] |
| 18 | CoS_x_-Hal/S | 1 | 0.0300% | [45] |
| 19 | FeHCF | 5 | 0.0240% | [46] |
| 20 | Co@NCNT-5 | 10 | 0.0058% | This work |

1. []J. Hafner. *J. Comput. Chem.*‌ **2008**, 29(13): 2044-2078. [↑](#endnote-ref-1)
2. []P. E. Blöchl. *PRB*. **1994**, 50(24): 17953. [↑](#endnote-ref-2)
3. [] J. P. Perdew, K. Burke, M. Ernzerh. *PRL*. **1996**, 77, 38653868. [↑](#endnote-ref-3)
4. []S. Grimme, S. Ehrlich, L. Goerigk. *J. Comput. Chem.*‌ **2011**, 32(7): 1456-1465. [↑](#endnote-ref-4)
5. []H. J. Monkhorst, J. D. Pack. *PRB*. **1976**, 13(12): 5188. [↑](#endnote-ref-5)
